# *C9orf72* Dipeptide Repeat Proteinopathy Is Linked to Increased Histone H3 Phosphorylation on Serine 10

**DOI:** 10.1021/acsomega.5c05836

**Published:** 2025-10-09

**Authors:** Samantha N. Cobos, Raven M. A. Fisher, Seth A. Bennett, Chaim Janani, David K. Dansu, Matthew M. Cleere, Arefa Yeasmin, Gabriel Cruz, Sidra Qureshi, William Villasi, Rania Frederic, Kyle Chen, Mila Mirzakandova, George Angelakakis, Elizaveta Son, Andrew Elgendy, Mariana P. Torrente

**Affiliations:** † Department of Chemistry and Biochemistry, 2037 Brooklyn College, Brooklyn, New York 11210, United States; ‡ Ph.D. Program in Chemistry, The Graduate Center of the City University of New York, New York, New York 10016, United States; § Department of Neurobiology and Behavior, Stony Brook University, Stony Brook, New York 11794, United States; ∥ Ph.D. Program in Biochemistry, The Graduate Center of the City University of New York, New York, New York 10016, United States; ⊥ Regeneron Pharmaceuticals, Inc., Rensselaer, New York 12144, United States; # Neuroscience Initiative, Advanced Science Research Center, CUNY, New York, New York 10031, United States; ∇ Regeneron Pharmaceuticals, Inc., Tarrytown, New York 10591, United States; ○ Ph.D. Program in Biology, The Graduate Center of the City University of New York, New York, New York 10016, United States; ◆ Structural Biology Initiative, Advanced Science Research Center, CUNY, New York, New York 10031, United States

## Abstract

Amyotrophic lateral sclerosis (ALS) and frontotemporal dementia (FTD) are fatal illnesses forming a neurodegenerative disease continuum. While most ALS/FTD cases are sporadic, a small proportion of cases are linked to mutations in many genes. Among these, hexanucleotide repeat expansions in the *C9orf72* gene are the most common and lead to the formation of dipeptide repeat proteins (DPRs), including a proline-arginine dipeptide (PR), which aggregate in the cytoplasm of decaying neurons. As genetics alone fails to explain the etiology of ALS/FTD, it is possible that epigenetic mechanisms – such as histone post-translational modifications (PTMs) – are involved in disease processes. A *Saccharomyces cerevisiae* (PR)_50_ overexpression model displays overt growth suppression and aggregation. Here, we exploit this model as a discovery platform to comprehensively characterize changes in the levels of PTMs on Histones H3 and H4. We find that overexpression of (PR)_50_ is associated with increased levels of phosphorylation on Histone H3 at Serine 10 (H3S10ph). Furthermore, (PR)_50_ overexpression revealed modest increases in the levels of other marks associated with increased gene expression. Remarkably, decreased abundance of Ipl1, the kinase responsible for phosphorylating H3S10 in yeast, leads to amelioration of the growth suppression phenotype and restores H3S10ph levels even in the context of (PR)_50_ overexpression. Recapitulating our results in yeast, several *c9orf72* ALS patient-derived fibroblasts and induced pluripotent stem cell (iPSCs) lines display similar increases in H3S10ph levels. Altogether, these findings reveal a previously undiscovered connection between H3S10ph and c9 ALS/FTD proteinopathy that could reveal novel targets for the treatment of this disease.

## Graphical abstract

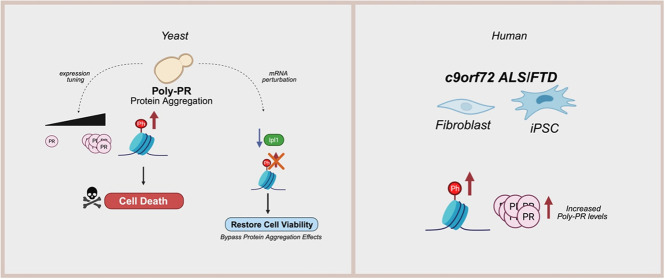

## Introduction

Amyotrophic lateral sclerosis (ALS) and frontotemporal dementia (FTD) are progressive, fatal diseases that lead to a plethora of debilitating symptoms.^1^ While each disease affects different neuronal types in distinct locations of the nervous system, ALS and FTD lie on a disease continuum sharing pathological pathways. Disease onset typically begins at age 60 and the probability of developing symptoms increases with age.^2^ The prognosis for ALS/FTD patients is very poor. At present, only a handful of FDA-approved medications exist to treat symptoms; however, available treatments fail to cure or stop the progression of the disease.^3,4^

The majority of ALS/FTD cases are sporadic, with only 5–10% of all cases running in families.^1^ Familial ALS/FTD is linked to mutations in many genes including *fused in sarcoma (FUS)*, *TAR DNA-binding protein 43 (TDP-43)*, and *chromosome 9 open reading frame 72 (c9orf72)*. Interestingly, the protein products of these mutant genes (FUS, TDP-43, and C9orf72, respectively) mislocalize to the cytoplasm of degenerating neurons and aggregate.^1,5,6^ Despite intense research, it is unclear how protein aggregation is linked to neuronal death.

The presence of a noncoding G_4_C_2_ hexanucleotide repeat expansion (HRE) in the *c9orf72* gene locus is the most common genetic cause of ALS/FTD.^7^ ALS/FTD patients present with 100s to 1000s of repeats in this gene, while healthy individuals have less than 30.^6^ This HRE can lead to the formation of toxic, aggregate-prone, abnormally transcribed RNAs that carry the repeat and form stable DNA and RNA structures called G-quadruplexes and R-loops.^8,9^ These RNAs can also undergo repeat-associated non-ATG (RAN) translation, leading to the production of five dipeptide repeat (DPR) proteins: glycine-proline (GP), proline-alanine (PA), glycine-alanine (GA), glycine-arginine (GR) and proline-arginine (PR).^10^ DPRs aggregate into insoluble neuronal inclusions throughout the central nervous system of c9ALS/FTD cases.^11^ While the discovery of *c9orf72* and the role that its products play in ALS/FTD has helped explain certain aspects of disease progression, a definitive cause for ALS/FTD has yet to be discovered. All in all, genetics alone fails to explain the occurrence of the disease. Hence, it is possible for epigenetic mechanisms to contribute to disease etiology.

Epigenetics is the study of heritable changes to an organism’s phenotype that take place without directly affecting its genome.^12^ These changes occur by way of regulating access to the genetic information. Eukaryotic DNA is packaged into chromatin, a highly organized protein–DNA complex. Chromatin can be found as either tightly wound, transcriptionally silent regions of heterochromatin, or loosely wound, transcriptionally active regions of euchromatin.^12^ The open structure of euchromatin allows for ease of access by transcriptional machinery to an organism’s DNA, allowing for gene transcription.^13^ Conversely, heterochromatin’s closed structure occludes gene accessibility, leading to transcriptional silencing.^14^ The basic unit of chromatin, termed the nucleosome, consists of DNA wrapped around an octameric histone protein core composed of two copies each of Histones H2A, H2B, H3, and H4. Epigenetic mechanisms such as DNA methylation and histone post-translational modifications (PTMs) can direct the formation of either euchromatin or heterochromatin and fine-tune gene accessibility in site-specific manners.^12,13^

Histone PTMs include chemical moieties such as mono-, di- or trimethylation, acetylation, and phosphorylation occurring in specific residues on the histone protein. The addition or removal of these PTMs is controlled by various histone modifying enzymes (HMEs) that can either “write” or “erase” the “code” comprised by these marks. “Reader” enzymes identify the presence/absence of certain PTMs and control gene accessibility to perform numerous cellular processes. For example, H3S10 can be phosphorylated by the “writer” Aurora B kinase, and is dephosphorylated by Protein Phosphatase 1 (PP1).^15,16^ H3S10ph is then “read” by a number of HMEs, such as the histone deacetylase HST2, responsible for modulating H4K16ac.^17^ Remarkably, histone PTMs, as well as their respective HMEs, are highly conserved across organisms.

Changes in the histone PTM landscape have begun to be characterized in ALS/FTD cellular and animal models, as well as patient samples.^18,19^ For instance, spinal cord samples from c9ALS/FTD patients display up-regulated levels of both γH2A.X (a histone mark highly conserved in its role as a marker of DNA damage) and phosphorylated kinase ataxia-telangiectasia mutated (ATM; the kinase responsible for phosphorylating H2A which also becomes phosphorylated following DNA damage), while c9ALS/FTD neuronal cells have increased γH2A.X levels.^20^ Moreover, frontal cortices and cerebella of c9ALS/FTD patients show increased levels of trimethylation of lysine residues on Histones H3 and H4.^21^ Furthermore, mutant *c9orf72* mice and cortices from c9ALS/FTD patients display increased levels of histone trimethylation.^22^ Lastly, increases in levels of H3K27me3 and H3K4me3 are linked to poly-PR repeats in c9ALS/FTD patients.^22^ Hence, c9 ALS/FTD connects to changes in histone PTMs.

Here, aiming for a more comprehensive characterization of the histone PTM landscape associated with poly-PR proteinopathy, we delineate the histone PTM landscape connected to c9 ALS/FTD by exploiting a poly-PR overexpression *Saccharomyces cerevisiae* model as a discovery platform. Yeast is a useful model for studying neurodegenerative diseases because it preserves many neuronal cellular pathways and it recapitulates proteinopathies and cytoplasmic foci seen in ALS/FTD upon DPR overexpression.^23^ Additionally, yeast and human Histones H3 and H4 share over 90% sequence homology, while corresponding HMEs remain highly conserved across organisms.^24^ Advantageously, overexpression of neurodegenerative proteinopathies in yeast leads to an easily detectable growth suppression phenotype,^23,25^ mimicking neuronal death and empowering us to expediently perform genetic manipulation experiments. Furthermore, mammalian models recapitulate three levels of mutant *c9orf72* toxicity (HRE, toxic RNA, and DPRs), leading to confounding variables and a nonspecific view of disease pathology. Yeast provides a unique platform to study individual aspects of toxicity, such as effects from DPRs alone, providing deeper insight into isolated facets of c9ALS/FTD. Yeast models using codon-optimized plasmids to produce DPRs without invoking HREs or toxic RNA have been developed, allowing for the study of peptide-specific pathways in the context of disease. Capitalizing on a (PR)_50_ overexpression yeast model,^23^ we characterize genome-wide changes in the levels of specific histone PTMs along the N-terminal tails of Histones H3 and H4. We find that many, but not all, lysine residues along the Histone H3 and H4 tails are hyperacetylated and hypermethylated in the context of (PR)_50_ overexpression. Notably, we find an important increase in levels of Histone H3 phosphorylation on Serine 10 (H3S10ph). We confirm that the magnitude of this increase is tied to the levels of (PR)_50_ expression. Moreover, disruption of the HME installing H3S10ph ameliorates the growth suppression phenotype and restores H3S10ph levels even in the context of robust (PR)_50_ overexpression and aggregation. Expanding these findings to human cellular c9ALS/FTD models, we probe for H3S10ph levels on histones isolated from c9ALS/FTD patient-derived fibroblasts and induced pluripotent stem cell (iPSCs) lines as well as age/sex matched controls. Excitingly, several cell lines also evidence a genome-wide increase in H3S10ph levels in c9ALS/FTD cells compared to controls. Overall, this is the first study connecting H3S10ph disruptions to c9ALS/FTD proteinopathy. Our results highlight the potential for novel epigenetic targets for the treatment of this disease and other neurodegenerative disorders.

## Results

### (PR)_50_ Overexpression in Yeast is Connected to Changes to the Histone Post-Translational Modification Landscape

Leveraging a previously developed yeast model overexpressing toxic dipeptide repeat products from mutant *c9orf72*,^23^ we uncovered changes in the epigenome connected to C9 proteinopathy. We created yeast strains harboring DPR plasmids; pAG303GAL-(PA)_50_, pAG303GAL-(GA)_50_, pAG303GAL-(GR)_100_, and pAG303GAL-(PR)_50_ (Figure 1). These strains are based on the W303 yeast background, and protein expression is induced by growth in galactose. As these constructs are codon-optimized to express DPRs without using the GGGGCC repetitive sequence, this model isolates the toxicity derived from protein aggregation rather than from RNA-based gain-of-function mechanisms. Furthermore, as yeast do not bear a *c9orf72* homologue, interference from native protein or loss of protein function mechanisms is not an issue. In agreement with previous reports,^23^ we find that (PA)_50_ and (GA)_50_ overexpression leads to normal growth while (PR)_50_ overexpression leads to strong growth suppression. (GR)_100_ overexpression, while slightly toxic, does not lead to the level of growth suppression that (PR)_50_ invokes (Figure 1A). We verified (PR)_50_ overexpression by dot blotting with a Poly-PR-specific antibody (Figure 1B). The pAG303GAL-PR_50_ plasmid includes both a FLAG and c-myc tag, which we used to further verify (PR)_50_ overexpression via Western Blot (Figure S1).^23^ Overexpression of (PA)_50_ and (GA)_50_ was also verified using immunoblotting for FLAG (Figure S2A,B). (GR)_100_ overexpression was verified with a Poly-GR antibody as the plasmid is untagged (Figure S2C). A dot blot, rather than a Western blot, was performed for GR as these are more generally used with DPR antibodies because potential protein aggregation can affect size separation. Of note, throughout our yeast experiments, we used pAG303GAL-ccdB as a vector control. The DNA gyrase inhibitor ccdB is used to enhance cloning efficiency by eliminating transformants containing nonrecombinant vectors in bacterial transformations; however, it is not functional in yeast.^26^ Thus, the ccdB vector serves as a control for the effects of transformation on the levels of histone PTMs in yeast.^27^ As (PR)_50_ overexpression led to the strongest growth suppression, we chose to focus our investigation on this DPR. As expected, there is no Poly-PR signal in yeast transformed with pAG303GAL-ccdB (Figure 1B). Additionally, no c-myc signal is detected in ccdB yeast, confirming that these yeasts do not bear the (PR)_50_ plasmid (Figure S1). We also verified the presence of (PR)_50_ aggregates in the cytoplasm of transformed cells by fluorescence microscopy using an anti-FLAG antibody (Figure 1C). We confirmed that no aggregates are present in the control yeast (Figure 1C,D).

**Figure 1:**
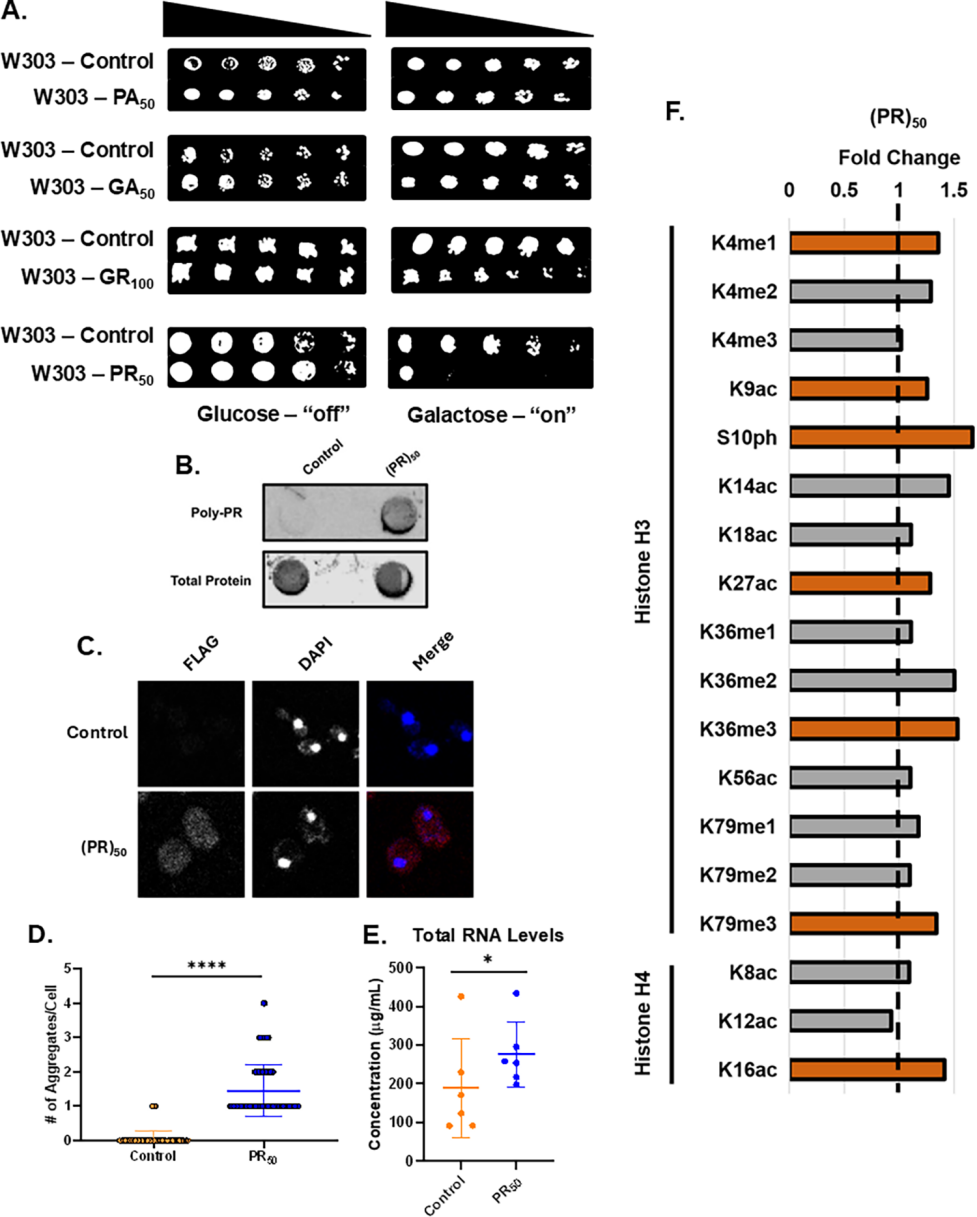
(PR)_50_ overexpression in W303 yeast leads to growth suppression and aggregation. (A) Solid media growth assay for yeast transformed with either a control vector (ccdB) or (PR)_50_ plasmid serially diluted and plated onto selective solid media supplemented with either glucose or galactose. (B) Dot blot showing levels of Poly-PR in (PR)_50_ yeast compared to the vector control (ccdB). Revert 700 total protein stain was used for normalization. (*n* = 3). (C) Confocal microscopy images of vector control (ccdb) and (PR)_50_ overexpression yeast staining for FLAG (red) and costained with DAPI (blue) (*n* = 3). (D) Column scatterplot depicts the number of aggregates per cell from either control (orange) or (PR)_50_ (blue) yeast. Each point represents a cell from each group. (*n* = 150–170), **** = *p* ≤ 0.0001. (E) Column scatterplot depicts concentrations (μg/mL) of purified RNA obtained from control (orange) and (PR)_50_ overexpression (blue) yeast. Each point represents a different biological replicate. (*n* = 6), * = *p* ≤ 0.05. (F) Bar graph depicting increases in relative densities of histone post-translational modification levels in (PR)_50_ yeast when compared to control yeast. Bar color depicts *p*-values for corresponding histone PTMs: *p* > 0.05 (gray); *p* ≤ 0.05 (orange), dashed line represents control for comparison.

Using previously published methods,^28^ we investigated the impact of the *c9orf72* DPR (PR)_50_ on the histone PTM landscape in yeast. Total protein lysates from (PR)_50_ as well as control yeast were subjected to immunoblotting probing for changes in levels of distinct histone H3 and H4 PTMs. Raw histone PTM signal intensities are normalized to raw histone H3 signal intensities to obtain relative density values for each experiment. It is important to note that different antibodies yield varying relative density readouts for reasons such as antibody sensitivity, sample loading, and transfer efficiency between experiments. As such, all immunoblot quantifications are normalized to Histone H3 total signal. We also verified linear range antibody response for relevant histone PTM antibodies (Figure S3). We focus on Histones H3 and H4 PTMs as these are the most pervasively modified. We also focused on the marks conserved from yeast to human.^12^ Surveying 18 histone PTMs, we find overexpression of (PR)_50_ in yeast is linked to genome-wide changes in levels of seven histone PTMs compared to controls (Figure 1F). Remarkably, changes in histone PTM levels occur as early as 8 h into protein overexpression. As such, all experiments were conducted with yeast harvested 8 h after galactose induction. To roughly assess if the histone PTM changes we find impact overall gene expression, we quantified total RNA levels from control and (PR)_50_ overexpression yeast. Intriguingly, we find that yeast overexpressing (PR)_50_ display an approximate 40% increase in levels of total RNA compared to control yeast (Figure 1E).

### (PR)_50_ Overexpression is Linked to Increases in the Levels of Histone H3 and H4 Acetylation at Specific Sites

We identify modest genome-wide increases in levels of H3K9ac, H3K14ac, H3K27ac, and H4K16ac in (PR)_50_ yeast compared to controls (Figure 2). Specifically, there is an approximate 25% increase in levels of H3K9ac and H3K14ac in (PR)_50_ yeast compared to control yeast (Figure 2A,B). In addition, there is an approximate 30% increase in levels of H3K27ac (Figure 2C) and a 50% increase in levels of H4K16ac in (PR)_50_ yeast when compared to controls (Figure 2D). Notably, not all acetylation sites were impacted. We find no statistically significant differences in the levels of H3K18ac, H3K56ac, H4K8ac, and H4K12ac in (PR)_50_ yeast when compared to controls (Figure S4). Altogether, the increase in the levels of acetylation marks (H3K9ac, H3K14ac, H3K27ac, and H4K12ac) found in (PR)_50_ overexpression yeast agrees with an increase in RNA levels and supports the notion of increased gene expression in this context (Figure 1E).

**Figure 2:**
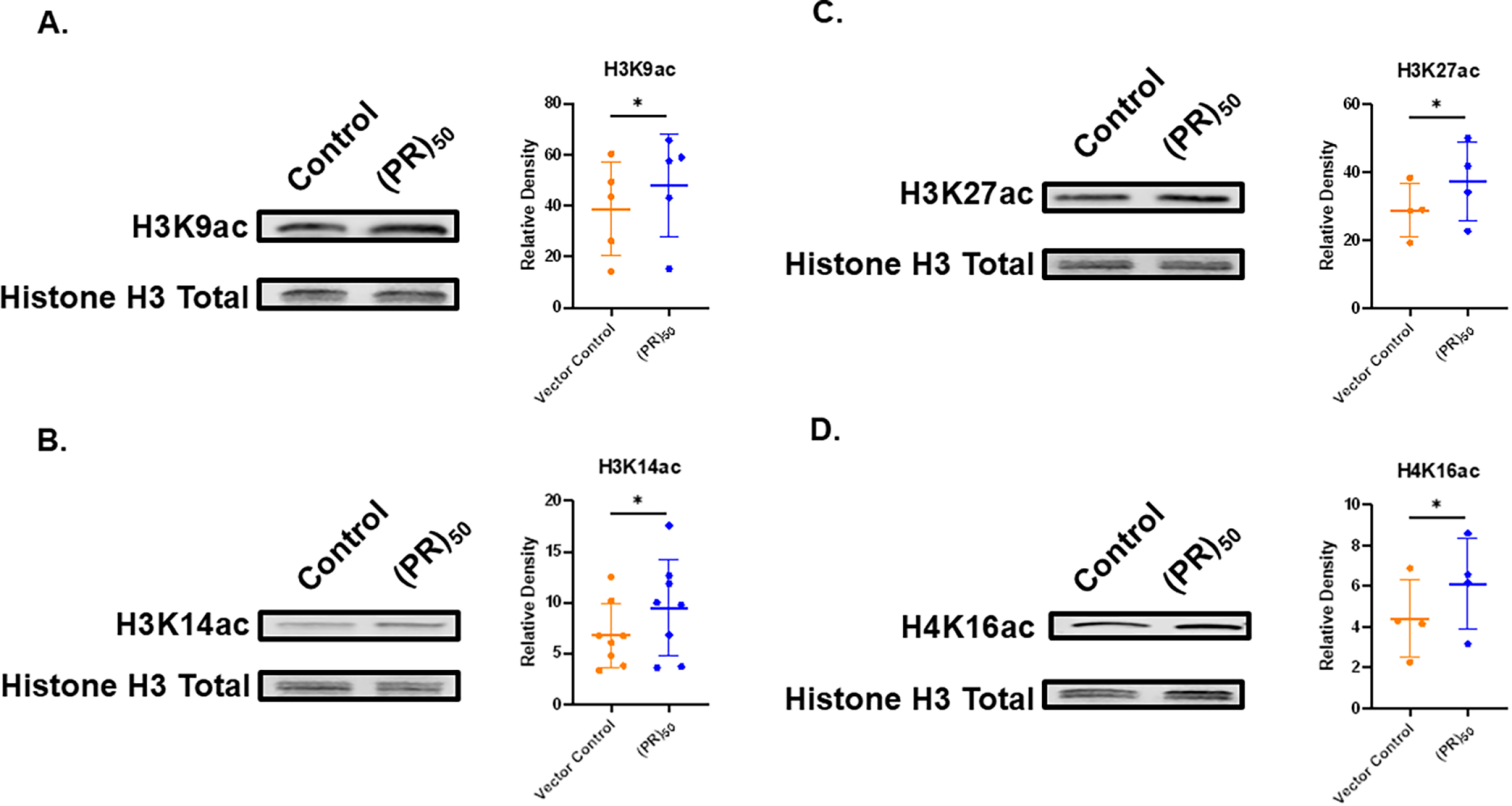
(PR)_50_ overexpression is linked to increases in acetylation on specific sites on Histones H3 and H4 in yeast. Representative blots showing increases in levels of (A) H3K9ac, (B) H3K14ac, (C) H3K27ac, and (D) H4K16ac in (PR)_50_ yeast compared to controls. Column scatterplots quantify the relative density levels of histone PTMs in (PR)_50_ yeast (blue) compared to loading controls (orange). Each point in the graph is a separate experiment with a different biological replicate. (*n* = 4–8), * = *p* ≤ 0.05.

### (PR)_50_ Overexpression is Linked to Increases in the Levels of Select Mono-, Di-, and Trimethylation Marks on Histone H3

Besides acetylation, we probed for changes in the levels of mono-, di-, and trimethylation on Histones H3 and H4. We find that (PR)_50_ overexpression is connected to changes on specific PTMs. We find moderate genome-wide increases in the levels of H3K4me1, H3K36me3, and H3K79me3 (Figure 3). In (PR)_50_ yeast, we find an approximate 30% increase in the levels of H3K4me1 (Figure 3A). Puzzlingly, we do not find a statistically significant change in the levels of H3K4me2 or H3K4me3 in (PR)_50_ yeast (Figure S5A,B). There is also a roughly 50% increase in levels of H3K36me3 and a 60% increase in levels of H3K79me3 (Figure 3B,C) in (PR)_50_ yeast compared to control. In contrast, levels of H3K36me1/me2, and H3K79me1/me2 show no statistically significant changes (Figure S5C–F). As H3K4me1, H3K36me3, and H3K79me3 are marks associated with active transcription,^29–31^ these data, together with increased RNA levels, further support increased gene utilization in (PR)_50_ yeast compared to controls.

**Figure 3:**
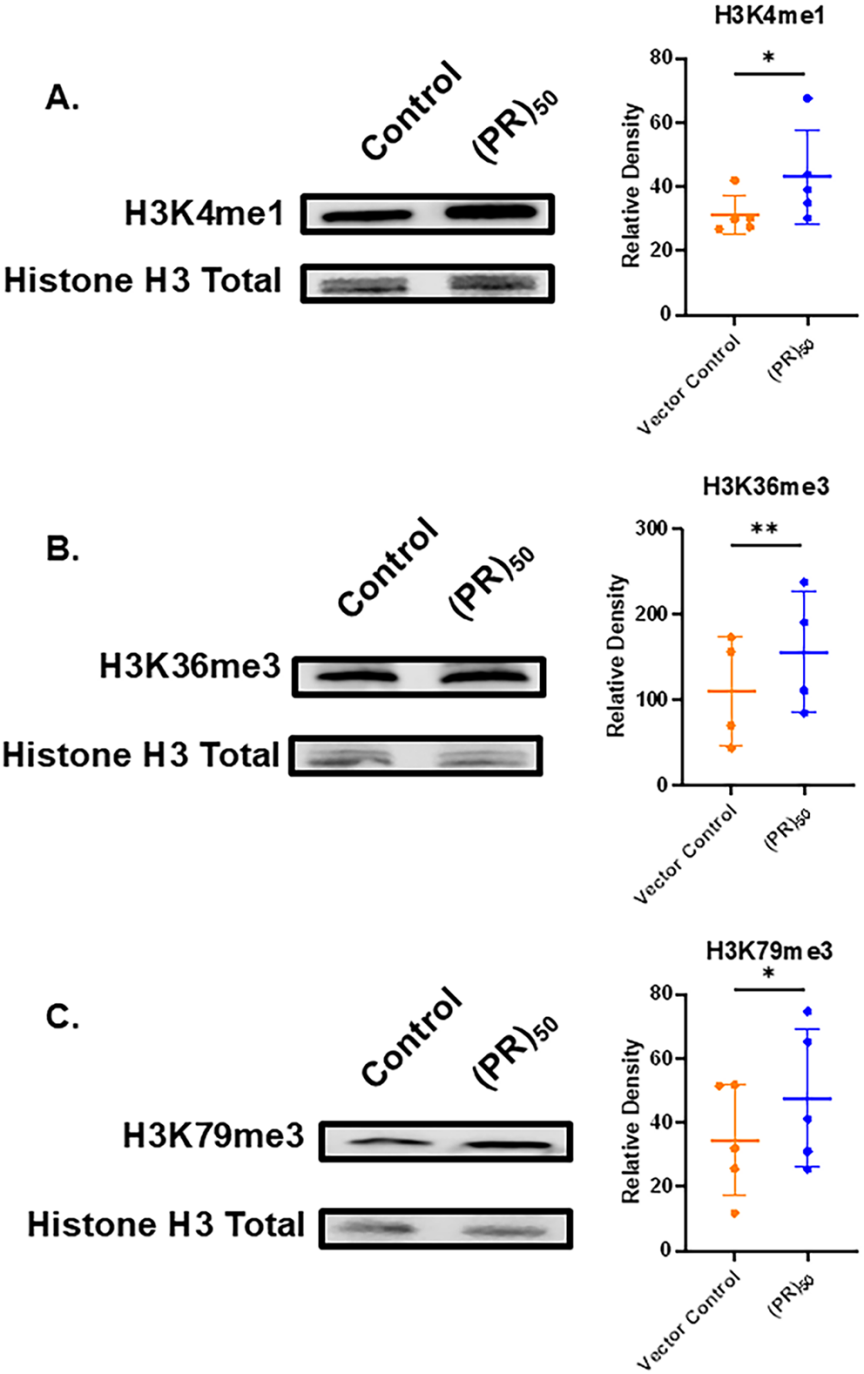
(PR)_50_ overexpression is associated with increases in the levels of specific methylation marks on Histone H3. Representative blots showing the levels of (A) H3K4me1, (B) H3K36me3, and (C) H3K79me3 in (PR)_50_ yeast compared to controls. Column scatterplots quantify the relative density levels of histone PTMs in (PR)_50_ yeast compared to loading controls. Each point in the graph is a separate experiment with a different biological replicate. (*n* = 4–5) * = *p* ≤ 0.05; ** = *p* ≤ 0.01.

### (PR)_50_ Overexpression is Connected to an Increase in the Levels of H3S10ph

Investigations into histone PTM in neurodegenerative disease typically focus on methylation and acetylation marks. Hence, histone phosphorylation has remained understudied in this context. Here, we find that overexpression of (PR)_50_ in yeast is linked to genome-wide changes in levels of H3S10ph compared to controls (Figure 1F). We identify a consistent 50% increase in the levels of H3S10ph in yeast overexpressing (PR)_50_ compared to controls (Figure 4A,B). We probed H3S10ph in yeast overexpressing other DPRs to explore the specificity of this increase. We do not find statistically significant changes in H3S10ph levels in yeast overexpressing (PA)_50_, (GA)_50_ or (GR)_100_ (Figure S2D–F).

**Figure 4:**
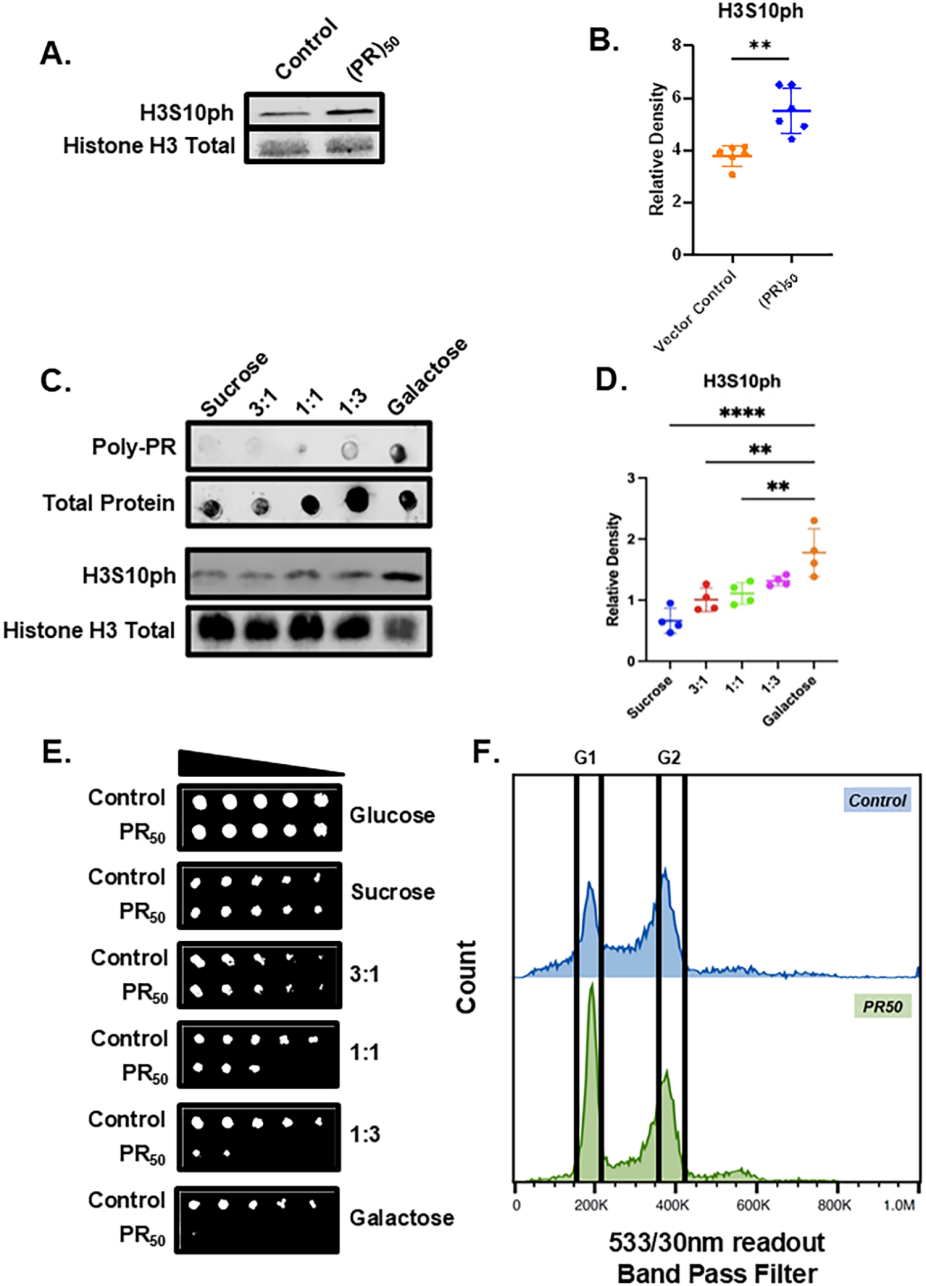
(PR)_50_ overexpression in yeast is connected to an increase the level of phosphorylation in Histone H3 at Serine 10. (A) Representative blots showing increases in levels of H3S10ph in (PR)_50_ yeast compared to controls. (B) Column scatterplots quantify the relative densities of raw H3S10ph signal to Histone H3 total signal from Panel A. Each point in the graph represents a biological replicate. (*n* = 6), ** = *p* ≤ 0.01 (C) Representative dot and Western blots showing changes in Poly-PR and H3S10ph in (PR)_50_ overexpression yeast grown in liquid culture supplemented with sucrose, galactose, and various ratios of sucrose:galactose. (*n* = 3–4 for 3 each experiment) (D) Column scatterplot representing relative levels of H3S10ph to Histone H3 total for each condition from Panel B. Each point in the graph is a separate experiment with a different biological replicate (*n* = 4) ** = *p* ≤ 0.01; **** = *p* ≤ 0.0001. (E) Solid media growth assay for yeast transformed with either a control vector (ccdB) or (PR)_50_ plasmid serially diluted and plated onto selective solid media supplemented with either sucrose, glucose, galactose, and various ratios of sucrose:galactose. (*n* = 4 for each condition). (F) Flow cytometry readout of control (ccdB; blue) and (PR)_50_ (green) yeast stained with SYTO 9 dye. Each experiment was performed three times with separate biological replicates.

To further establish that increases in H3S10ph levels are tied to (PR)_50_ overexpression, we “tuned” (PR)_50_ overexpression by growing yeast in varying ratios of sucrose:galactose (Figure 4C–E). Sucrose is metabolized to glucose, which then represses the galactose promoter responsible for (PR)_50_ overexpression.^32^ First, we verified that the sucrose:galactose setup reduced (PR)_50_ expression. Indeed, growth in sucrose nearly abolished (PR)_50_ expression when compared to growth in galactose (Figure 4C). Unsurprisingly, decreased expression of (PR)_50_ lessened the toxic phenotype (Figure 4E). As the concentration of galactose increased, the levels of H3S10ph in (PR)_50_ yeast also increased (Figure 4C,D). Correspondingly, we observe that lowering the level of (PR)_50_ resulted in a decreased magnitude of change in the level of phosphorylation of histone H3 on serine 10 (Figure 4C,D).

Given the increase in H3S10ph, a potent marker of mitosis,^33^ we considered the possibility that H3S10ph changes could arise from cell cycle arrest elicited by protein overexpression. To determine if this was the case, we performed flow cytometry experiments on control and (PR)_50_ yeast (Figure 4F). While (PR)_50_ cells have sharper peaks than controls, we find that the population of cells within each cell cycle phase is similar. The broad peaks displayed by control cells are due to a larger spread of fluorescent signals arising from variation within living cells. All groups of cells demonstrated no significant difference in the number of cells in G1 versus G2 stages, evidencing that H3S10ph level changes in (PR)_50_ yeast do not result from cell cycle disturbances. Full triplicate flow cytometry data is shown in Figure S6.

To confirm that the H3S10ph change is specific to (PR)_50_ toxicity and not a result of overexpression and/or protein aggregation toxicity in general, we assessed H3S10ph levels in a TDP-43 overexpression model.^25^ While overexpression of TDP-43 correlates to a strong growth suppression phenotype, we find that it results in no significant difference in levels of H3S10ph when compared to controls (Figure S7), suggesting that the increase in H3S10ph levels in (PR)_50_ yeast is specifically connected to overexpression of this polypeptide, and not general protein aggregation pathways.

### Decreased Abundance by mRNA Perturbation (DAmP) of Ipl1 Restores Normal H3S10ph Levels and Growth in (PR)_50_ Yeast

In humans, phosphorylation of H3S10 is installed by Aurora B kinase.^34^ In yeast, this modification is installed by Ipl1, the yeast homologue of Aurora B.^34^ As levels of H3S10ph are increased in (PR)_50_ yeast, this suggests that (PR)_50_ overexpression could be linked to enhanced Ipl1 activity, driving increased H3S10ph levels. Hence, reduced Ipl1 activity might result in the restoration of cell viability and H3S10ph levels. The ease of genetic manipulation makes yeast an ideal model to easily test this hypothesis. While Ipl1 is an essential gene and cannot be entirely knocked out, mRNA perturbation provides a way to reduce its expression. Commercially available Ipl1 Decreased Abundance by mRNA Perturbation (DAmP) strains (henceforth referred to Ipl1 DAmP) were transformed with both control and (PR)_50_ plasmids. It is important to note that our original experiments were performed in the W303 strain background (full genotype in Experimental Procedures), while these knockdowns are available in the BY4741 background (full genotype in Experimental Procedures). As such, we also included parental BY4741 strains in this set of experiments to serve as controls displaying normal Ipl1 activity.

Serial growth assays of BY4741 control and (PR)_50_ yeast reveal that the toxic growth defect caused by (PR)_50_ overexpression also occurs in this genetic background (Figure 5A). We verified (PR)_50_ expression (Figure 5B–E). As expected, BY4741 (PR)_50_ yeast also displayed a 50% increase in levels of H3S10ph compared to BY4741 control yeast (Figure 5F,G). Strikingly, serial dilution growth assays of Ipl1 DAmP yeast show marked improvement in growth despite robust (PR)_50_ overexpression and aggregation (Figure 5A,B). Immunoblots revealed no statistically significant changes in levels of H3S10ph between Ipl1 DAmP control and (PR)_50_ cells, indicating correction of H3S10ph levels (Figure 5F,H).

**Figure 5:**
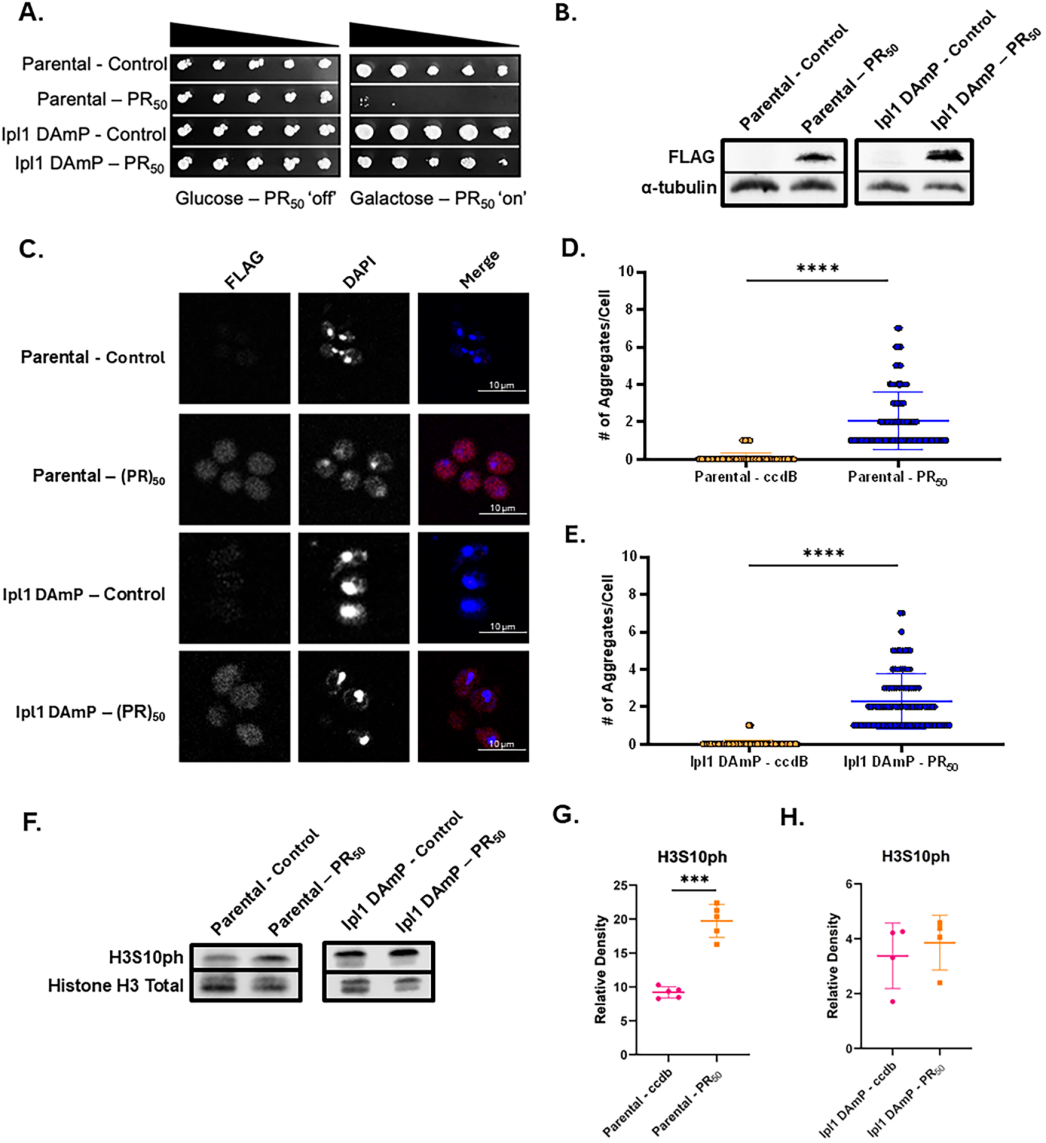
mRNA perturbation of Ipl1 leads to amelioration of growth suppression and restores H3S10ph levels in (PR)_50_ overexpression yeast. (A) Serial growth assay verifying toxicity elicited by (PR)_50_ overexpression in BY4741 (Parental) and Ipl1 DAmP yeast. (B) Western blot verifying presence of (PR)_50_ in parental and Ipl1 DAmP (PR)_50_ yeast. (C) Confocal microscopy of parental and Ipl1 DAmP control and (PR)_50_ yeast stained with α-FLAG (red) and a DAPI (blue) costain. (D) Column scatterplot depicts the number of aggregates per cell from either parental control (orange) or (PR)_50_ (blue) yeast. Each point represents a cell from each group. (*n* = 149–152), **** = *p* ≤ 0.0001. (E) Column scatterplot depicts the number of aggregates per cell from either Ipl1 DAmP control (orange) or (PR)_50_ (blue) yeast. Each point represents a cell from each group. (*n* = 102–111), **** = *p* ≤ 0.0001. (F) Western blot of parental and Ipl1 DAmP control and (PR)_50_ yeast probing for H3S10ph and Histone H3 Total. (G) Column scatterplot quantifies the relative density of H3S10ph levels in parental control and (PR)_50_ yeast. Each point in the graph represents a separate experiment with a different biological replicate. (*n* = 5; *** = *p* ≤ 0.001). (H) Column scatterplot quantifies relative density of H3S10ph levels in Ipl1 DAmP control and (PR)_50_ yeast. Each point in the graph represents a separate experiment with a different biological replicate. (*n* = 5; *** = *p* ≤ 0.001).

To assess the specificity of this connection, we verified that Ipl1 knockdown has no effect on the toxicity of TDP-43 overexpression (Figure S8A,B). Furthermore, immunoblots of both parental and Ipl1 DAmP TDP-43 cells revealed no statistically significant changes in H3S10ph levels (Figure S8C,D).

### C9orf72-Based ALS/FTD Male Patient-Derived Fibroblasts Exhibit an Increase in Levels of H3S10ph

Extending our findings into human cellular models, we probed for changes in H3S10ph in c9orf72–ALS (c9ALS) patient-derived fibroblasts compared to age/sex matched controls. Patient-derived fibroblasts are efficient and economical models that recapitulate disease features found in neurons.^35,36^ H3S10ph levels in purified histones were analyzed by immunoblotting. We analyzed histones from three male and three female patients alongside their respective controls. Of note, cell line pairs A and B shared the same age and sex matched healthy patient sample as a control, while pairs C and D shared the same age and sex matched sample as a control. Recapitulating the findings in the yeast (PR)_50_ overexpression model, we find an approximate 25% increase in H3S10ph levels in fibroblasts from male patients harboring the HRE *c9orf72* mutation compared to controls (Figure 6A; cell line pairs A, B, and F). Cells from female patients, however, show no statistically significant change in levels of H3S10ph when compared to controls (Figure 6A; cell line pairs C, D, and E). Since we found differential H3S10ph levels in our fibroblast groups, we explored Poly-PR levels in one patient pair with increased H3S10ph, cell line pair F, and one group without increased H3S10ph, cell line pair D. We find no significant increases in Poly-PR levels in cell line pair F, but pair D does have lower PR levels than pair F (Figure S9). Overall, exploring DPR levels in these fibroblast pairs is an avenue for further investigation. Interestingly, two c9ALS/FTD patient-derived induced pluripotent stem cell (iPSC) lines matched to isogenic controls show slightly contrasting results. While both iPSC cell groups are derived from male patients, one shows no statistically significant difference in H3S10ph levels (Figure S10A), However, a second iPSC pair shows displays a 50% increase in H3S10ph levels when compared to controls, akin to (PR)_50_ yeast and the male fibroblast pairs (Figure S10B). Interestingly, the iPSC group that reveals increases in H3S10ph also displays increased Poly-PR levels while the other iPSC group does not (Figure S10C,D). A full list of all human cell lines used is detailed in Table S1.

**Figure 6:**
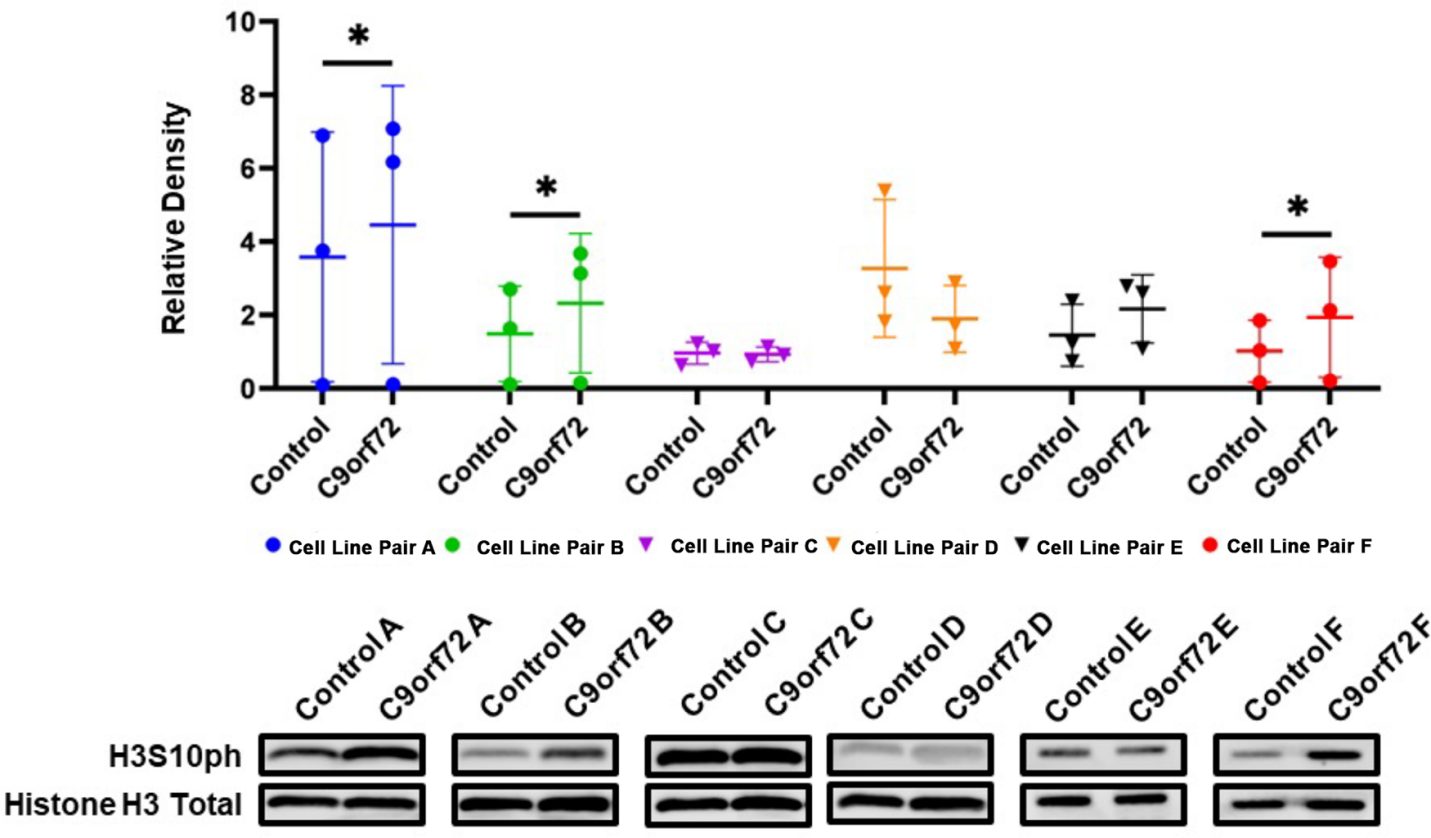
Genome-wide H3S10ph levels are increased in male c9orf72-based ALS patient-derived fibroblasts. (A) Representative blots probing for changes in H3S10ph in isolated histones from c9ALS patient-derived fibroblasts. Cell line pairs A, B, and F are derived from male patients, while cell line pairs C, D, and E are derived from female patients. Controls are age/sex matched. Column scatterplots quantify the relative densities of raw H3S10ph signal to Histone H3 total signal. Each point in the graph represents a separate experiment with a different biological replicate. (*n* = 3), * = *p* ≤ 0.05.

## Discussion

Findings in the past decade have highlighted epigenetic channels as a potential route for neurodegenerative disease therapy.^37–40^ *S. cerevisiae* models are a valuable platform to launch epigenetic neurodegeneration studies for their versatility, conservation of epigenetic mechanisms, overt phenotype, cost-effectiveness, and ease for genetic manipulation. Here, we exploited a (PR)_50_ overexpression yeast model to reveal that a toxic C9ALS/FTD dipeptide repeat is linked to changes in the histone PTM landscape. We have previously exploited similar models to examine histone modifications in the context of FUS and TDP-43 proteinopathies.^25^ Through immunoblotting techniques, we discover genome-wide increases in the levels of H3K4me1, H3K9ac, H3K14ac, H3K27ac, H3K36me3, H3K79me3, H4K16ac, and H3S10ph are connected to (PR)_50_ overexpression in yeast (Figure 1). Notably, these variations detected genome-wide are likely a significant underestimate of the potential greater differences detected at specific chromatin loci. Our findings agree with and expand upon previous work revealing that trimethylated lysine residues on the tails of histones H3 and H4 bind strongly to *c9orf72* expanded repeats in brain tissue, but not to nonpathogenic repeats, suggesting changes to the histone PTM landscape occur in the context of C9ALS.^21^

Overall, increased histone acetylation is an indicator of open and accessible sites of chromatin.^41–45^ Acetylation of certain residues have has more unique functions; for instance, increased levels of H4K16ac promotes activation of the ATM kinase, a key player in sensing DNA damage.^46,47^ Meanwhile, acetylation of H4K8 and H4K12 are critical for chromatin decompaction during DNA replication.^48^ H3K9ac is also altered during the DNA replication process as an effect of supercoiling stresses.^49^ The *c9orf72* HRE is associated with increased instability and decreased DNA replication stability, suggesting a possible reason why these marks are affected by (PR)_50_ overexpression.^50^ Methylation of histone tails invokes a wider breadth of functions. H3K4me1 is observed across most active genes.^51^ H3K36me3 can play several roles, such as serving as a platform for HDACs to then deacetylate histones^52,53^ and serving as a mark of histones that have been displaced by RNA polymerase II during transcription.^54^ H3K79me3, like H3K4me1, is observed in diverse active genes, and is also involved in transcriptional activation and elongation, and DNA damage response.^31,55^

H3K36me3 is also required for homologous recombinational repair of DNA damage when deposited with H4K16ac.^56–58^ Intriguingly, many of the marks that change in the context of (PR)_50_ overexpression point to a potential role for DNA damage and repair, while also signaling for gene activation.^59–61^ Abundant DNA damage is also associated with global levels of genomic instability and overall chromatin decondensation.^62^ Could the increase in DNA damage-associated PTMs allude to dysregulation caused by (PR)_50_ overexpression? Indeed, previous literature has identified that U-2 OS cells transfected with either poly-PR, poly-GR, or poly-GA experience decreased efficiency of several DNA damage repair mechanisms.^63^ Furthermore, *c9orf72-*deficient neurons experienced attenuated nonhomologous end joining repair,^64^ suggesting that normal *c9orf72* plays a role in nonhomologous end joining that is lost in patients with the GGGGCC mutation. Overall, defects in DNA damage repair are predominant in the context of C9ALS/FTD, and the findings presented here in yeast could reveal more information about how DPRs affect cellular processes such as DNA damage repair.

Importantly, we also identify a sharp and reproducible increase in genome-wide- levels of H3S10ph in (PR)_50_ yeast compared to controls (Figure 4). Yeast expressing other DPRs do not display such increase (Figure S2). Evidence from others also hints at an important role for H3S10ph in *c9orf72* pathology. H3S10ph is involved in the formation of euchromatin through the displacement of heterochromatin protein 1 from chromatin.^33^ *C9orf72* expansions have been linked to defects in heterochromatin.^22^ In particular, poly-PR was found to interact with heterochromatin in patient post-mortem tissues. Poly-PR was also associated with aberrant histone methylation profiles and was found to adversely influence heterochromatin structure.^22^ Furthermore, elevated levels of RNA/DNA hybrids (R-loops) were found in rat cortical neurons and MRC5 human fetal lung fibroblasts transfected with either *c9orf72* RNA repeat expansion foci or poly­(glycine-arginine) DPRs, as well as c9 ALS patient spinal cord tissues.^9^ R-loops have an association with H3S10ph and chromatin condensation, thus, it is likely that H3S10ph is enhanced at the *c9orf72* locus.^65^ More recently, poly-PR has been connected to H3S10ph alterations leading to the increased expression of the stress granule protein G3BP1 in fly and cellular models.^94^ Taken together, these data raise the hypothesis that poly-PR alters heterochromatin structure and gene expression potentially through H3S10ph dysregulation, but further research is needed to definitively establish such a mechanism.

H3S10ph is installed by Aurora B kinase in humans, or its homologue Ipl1 in yeast.^34^ This mark is removed by either PP1 (protein phosphatase 1) in humans or Glc7 in yeast.^16^ As increases in H3S10ph are tied to (PR)_50_ expression (Figure 4), we hypothesized that lowering Ipl1 activity could restore H3S10ph levels and ameliorate (PR)_50_’s toxicity. Yeast offers a facile and expedient model to test this hypothesis. Through the use of a yeast strain bearing decreased Ipl1 levels, we were able to show that decreased levels of Ipl1 not only restored H3S10ph levels in the context of (PR)_50_ overexpression but also rendered yeast impervious to (PR)_50_ overexpression toxicity (Figure 5). These results suggest that epigenetic manipulation can bypass the detrimental effects of protein aggregation. Additionally, our data suggest that Ipl1 and H3S10ph are involved in the mechanisms connecting (PR)_50_ overexpression and aggregation to cell death.

Several neurodegenerative disease models have shown evidence that the presence of protein aggregates is associated with defects in nucleocytoplasmic transport.^66,67^ A Drosophila model recapitulating the generation of toxic RNA products and DPRs from the *c9orf72* HRE found deficits in the nuclear pore complex in fly salivary glands.^68^ This leaves an open possibility of (PR)_50_ overexpression leading to similar deficiencies in the yeast model studied here. A recently developed coarse-grained molecular dynamics model reveals that poly-PR can bind directly to various nuclear transport components.^69^ Specifically, (PR)_50_ was found to bind to RanGAP, a vital member of both nuclear import and export cycles.^69,70^ Thus, poly-PR aggregates could dysregulate vital import/export sites at the nuclear pore complex, preventing Ipl1 from traveling between the cytoplasm and nucleus. Increased H3S10ph levels could indicate that Ipl1 may reside in the nucleus longer than usual (Figure 7, green). An alternative possibility involves the sequestration of Glc7, the phosphatase responsible for dephosphorylating H3S10, resulting in increased H3S10ph levels. All *c9orf72*’s DPR products mislocalize and aggregate in the cytoplasm of patient motor neurons.^7^ As they form aggregates, they are prone to sequestering other proteins in the cytoplasm, disrupting normal biological processes. It is plausible that (PR)_50_ aggregates could retain Glc7 in the cytoplasm (Figure 7, blue), increasing H3S10ph levels by impeding the removal of the modification. This could be confirmed by immunofluorescence, however, there is a severe lack of yeast-specific antibodies, thus complicating this type of study. Interestingly enough, overexpression of Sds22, a regulatory subunit of Glc7, leads to enhanced (PR)_50_ toxicity in this same yeast model,^23^ further suggesting that (PR)_50_ disrupts normal H3S10 phosphorylation patterns and regulation. Our results support the notion that Ipl1 and H3S10ph contribute to the growth suppression observed in (PR)_50_ yeast and highlight the potential for inhibiting Aurora B kinase activity as a therapeutic option for C9ALS/FTD. In fact, Aurora B inhibition was reported to enhance mitochondrial transport in iPSC-derived neurons from an ALS patient.^65^

**Figure 7:**
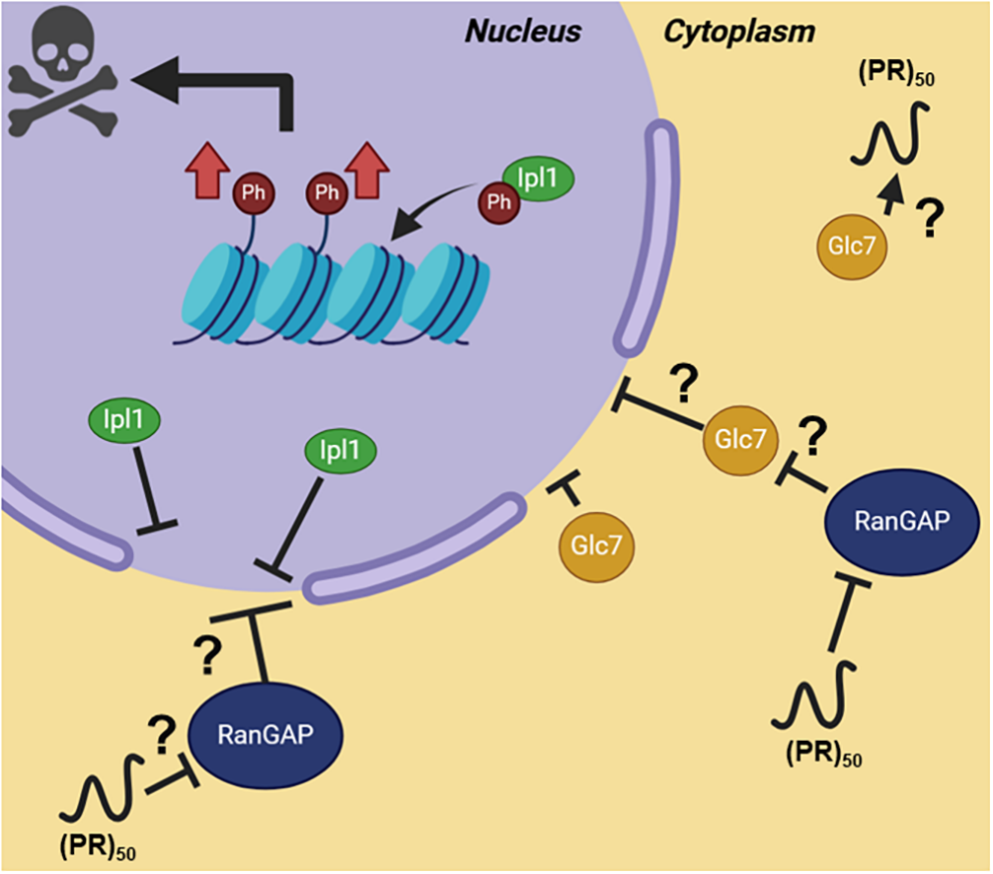
Overexpression of (PR)_50_ in yeast drives hyperphosphorylation of H3S10 through dysregulation of Ipl1. (PR)_50_ polypeptides (black) are connected to both global increases in levels of H3S10ph and a growth suppression phenotype when overexpressed in yeast. Both effects can be modified by modulating Ipl1 (green), suggesting that (PR)_50_’s toxicity is connected to epigenetic pathways. (PR)_50_ possibly inhibits RanGAP, thus dysregulating nucleocytoplasmic export of Ipl1 or Glc7 (yellow). Created with BioRender.com.

Importantly, the yeast model we exploit here only mimics the effects of the poly-PR dipeptide repeat protein, while patient neurons experience the effects of (1) the loss of function of *c9orf72*, (2) the toxic RNA repeats from the mutant gene, and (3) the effects of five possible dipeptide repeats, all with varying peptide lengths. To probe whether human cells recapitulate the H3S10ph alterations observed in yeast, we examined H3S10ph levels in C9ALS patient-derived fibroblasts. Fibroblasts have been used as model systems for ALS/FTD and are easy to grow in large numbers.^71,72^ Interestingly, some fibroblast lines recapitulated H3 methylation patterns found in *c9orf72* brains.^21^ In this study, we find a 25% increase in the levels of H3S10ph in fibroblasts derived from patients bearing *c9orf72* repeat expansions compared to age/sex-matched controls (Figure 6). Surprisingly, patient-derived fibroblast results suggest a sexual dimorphism. Male C9ALS/FTD fibroblasts displayed an increase in levels of H3S10ph as in the yeast model, however female C9ALS/FTD fibroblasts showed no such change (Figure 6). Puzzlingly, there is a higher incidence of female patients bearing *c9orf72*-related ALS, but no difference in terms of incidence of *c9orf72*-based FTD between the sexes.^73^ Nevertheless, while the sex-based bias in our fibroblast data was unexpected, it is not unprecedented. Primary human fibroblasts from eosinophilic esophagitis patients revealed statistically significant changes in gene expression in cells from male patients when compared to female patients.^74^ Additionally, histones from male-derived iPSCs with isogenic controls exhibit conflicting results, with one line displaying H3S10ph increases, while another does not (Figure S10A,B). It is also possible that discrepancies in the levels of H3S10ph do not arise from sex differences, but rather from heterogeneity in DPR distribution among lines.^75,76^ While the data hints at differential DPR accumulation in fibroblasts, further work is needed to thoroughly characterize these species (Figure S9). However, in iPSC groups, we have connected increased H3S10ph with high Poly-PR levels (Figure S10). Moreover, we were initially surprised to find any aberrant histone profiles in undifferentiated iPSC lines, as we expected the reprogramming process to erase most epigenetic signatures. We hypothesize that H3S10ph alterations result from epigenetic variation in the reprogramming process or from intrinsic epigenetic signatures related to disease that do not get erased by reprogramming.^77^ Overall, our findings in human cells further highlight H3S10ph as a novel target in C9ALS/FTD.

This study is still with limitations. First, as mentioned before, yeast models only show changes to the histone PTM landscape that are associated with the overexpression of (PR)_50_, which represents only a single facet of C9ALS/FTD. The use of patient-derived fibroblasts and iPSCs addresses this problem, but only to a certain extent. Ultimately, C9ALS/FTD affects motor neurons and the cerebral cortex,^7^ models yet to be probed in the manner presented here. Alas, obtaining a large enough number of human neurons for epigenetic studies is both technically challenging and expensive. Second, we only probe genome-wide changes to the epigenome. As such, our experiments do not allow for the detection of changes to the histone PTM landscape at specific genomic loci. It is likely that many of the modification changes we detect are even larger in magnitude at specific genomic loci. It is also likely that we are overlooking histone PTM changes that are only detectable on certain genes. For instance, altered levels of trimethylation of lysine residues on histones H3 and H4 were detectable only around the *c9orf72* hexanucleotide repeat expansion sites.^21^ A potential approach to overcome this would be the use of chromatin immunoprecipitation against H3S10ph in patient-derived neurons, as well as fibroblasts and iPSCs, to further elucidate the impact of mutant *c9orf72* on the epigenetic landscape. Third, our studies do not delineate mechanistic details connecting C9 proteinopathy and the epigenome. Subsequent studies will aim to understand the underlying mechanisms ultimately driving changes to the histone PTM landscape. What are the direct (or indirect) interactions that allow for changes to the histone PTM panorama? Lastly, it is difficult to establish causal relationships from our experiments: do changes in histone PTMs lead to disease processes, or do disease processes lead to changes in histone PTMs? Issues of causality are a concern whenever discussing histone modifications in general.^78^ Regardless, we have established a clear association of changes in histone methylation, acetylation, and phosphorylation with C9 proteinopathy. Even if histone PTMs are a consequence of disease processes, histone modification pathways are dynamic and pharmacologically accessible and able to impact cellular outcomes, and thus, they hold great promise for disease treatment regardless of causation.

Altogether, our data represents evidence for an association between altered levels of H3S10ph and DPR proteinopathy. Overexpression of (PR)_50_ is linked to changes in genome-wide levels of histone post-translational modifications in yeast. These changes are specific to (PR)_50_ proteinopathy. Remarkably, we show that we can bypass protein aggregation toxicity via genetic manipulation of histone modifiers. We have also identified a significant increase in the levels of H3S10ph in C9ALS/FTD patient-derived fibroblasts and iPSCs as well as a link between Poly-PR levels and H3S10ph increases in iPSCs, further suggesting a role for H3S10ph dysregulation in the etiology of C9ALS/FTD. These experiments represent progress toward identifying Aurora B kinase as a novel target for C9ALS/FTD therapeutic development. We hope that these findings ultimately lead to the development of H3S10ph and other histone marks as novel biomarkers for C9ALS/FTD and other neurodegenerative disorders.

## Experimental Procedures

### Materials

All chemicals are from Sigma-Aldrich (St. Louis, MO) unless otherwise specified.

### Yeast Strains and Plasmids

Histone PTM changes were probed in W303a yeast (*MATa*, *can1–100*,*his3–11*,15,*leu2*,*3 11*,12,*trp1–1*,*ura3–1*,*ade2–1*).^79^ Genetic inhibition experiments were conducted in BY4741 and Ipl1 DAmP yeast (*MATα his3Δ1 leu2Δ0 ura3Δ0 met15*Δ*0)* obtained from Horizon Discovery (Waterbeach, U.K.).^80^ The DPR plasmids; (PA)_50_ (pAG303GAL-PA50), (GA)_50_ (pAG303GAL-GA50), (GR)_100_ (pAG303GAL-GR100), and (PR)_50_ (pAG303GAL-PR50) were a gift from A. Gitler (Addgene plasmids no. 84906, 84907, 84908 and 84905).^23^ A control ccdB plasmid, pAG303GAL-ccdb was a gift from S. Lindquist (Addgene plasmid no. 14133). The TDP-43 plasmid (pAG303GAL-TDP-43) was a gift from Jackrel and Shorter.^81^ Yeast was transformed using standard poly­(ethylene glycol) and lithium acetate protocols.^28^

### Yeast Culture

Prior to all experiments, either W303a or BY4741 yeast were streaked onto YPD and incubated at 30 °C for 2–3 days (Ipl1 DAmP yeast were streaked onto YPD + 200 μg/mL G418). Strains were then inoculated into YPD liquid media (or YPD + 200 μg/mL G418 in the case of Ipl1 DAmP strains) and grown to saturation overnight at 30 °C with shaking at 200 rpm. Liquid cultures were then standardized to an OD_600_ of 0.3 and allowed to grow for 5 h at 30 °C at 200 rpm until an OD_600_ of 0.6–0.8 was reached.^28^ Yeast were transformed using standard poly­(ethylene glycol) and lithium acetate protocols.^28^ Transformed W303a and BY4741 yeast were grown in synthetic dropout medium lacking histidine (-His) supplemented with 2% of either glucose, galactose, or raffinose. Transformed Ipl1 DAmP yeast were grown in synthetic dropout medium lacking histidine (-His) supplemented with 2% of either glucose, galactose, or raffinose, along with 200 μg/mL G418. Cells were then pelleted, flash frozen, and stored at −80 °C.

### Solid Media Growth Assays

Yeast strains were grown to saturation overnight in raffinose-supplemented dropout media at 30 °C. For Ipl1 DAmP strains, G418 (200ug/mL) was added to the media. Overnight cultures were diluted 2-fold, then serially diluted 5-fold. A volume of 2 μL for each dilution was pipetted onto synthetic dropout agar plates supplemented with either glucose or galactose (200ug/mL G418 was added to media for Ipl1 DAmP strains). Plates were analyzed after 2–3 days of growth at room temperature. All experiments were repeated a minimum of three times with three independently transformed yeast strains.

### Protein Overexpression

Transformed yeast strains were grown to saturation overnight in raffinose-supplemented dropout media at 30 °C and 200 rpm. Overnight cultures were then diluted to an OD_600_ of 0.30 in galactose-supplemented synthetic dropout media to induce DPR overexpression and grown for 8 h at 30 °C at 200 rpm. Yeast cultures were then standardized to the lowest OD_600_. Cells were pelleted by centrifugation at 800 rcf for 5 min at 4 °C and washed three times with sterile water. Pellets were then flash frozen and stored at −80 °C. DPR presence was probed through Western or dot blot.

### RNA Purification and Quantification

RNA purification and quantification was performed as previously described.^82^ Frozen yeast pellets were thawed and cell counts were normalized upon counting with a hemocytometer (Thermo Fisher, Waltham, MA; cat. no. 501311352). Cells were then treated with 100 units of Zymolyase-20T (Nacalai USA, San Diego, CA; cat. no. 07663–91) for 30 min at 30 °C. RNA was isolated using a RNeasy Mini Kit from Qiagen (Germantown, MD; cat. no. 74104) according to the manufacturer’s instructions. Total RNA levels were measured in a Qubit 2.0 Fluorometer (Thermo Fisher Scientific), using a Qubit RNA Broad Range (BR) Assay Kit (Thermo Fisher Scientific, cat. no. Q10210). All experiments were repeated a minimum of three times with independent cell samples.

### Western Blotting

Frozen yeast pellets were thawed and processed for Western Blotting as previously described.^28^ Briefly, cell pellets were treated with 0.2 M NaOH and β-mercaptoethanol for 10 min on ice, pelleted again, and resuspended in 100 μL of 1× SDS sample buffer. Samples were then boiled at 95 °C for 10 min, followed by separation of cell lysates by SDS-PAGE using a 15% polyacrylamide gel and transfer to a PVDF membrane (EMD Millipore, Taunton, MA). Membranes were blocked using LI-COR blocking buffer (LI-COR Biosciences, Lincoln, NE) for 1 h at room temperature with gentle rocking. Primary antibody incubations were performed at 4 °C overnight with rocking. For histone modification detection, antibody names, manufacturer, catalogue number, and dilutions can be found in Table S2. Blots were processed using goat antimouse and goat antirabbit secondary antibodies (LI-COR Biosciences) and imaged on an Odyssey Fc Imaging System (LI-COR Biosciences). All experiments were performed a minimum of three times with independent cell samples.

### Immunocytochemistry and Confocal Microscopy

(PR)_50_ and ccdB control transformed W303a, BY4741, and Ipl1 DAmP yeast were imaged using a standard protocol.^83^ Briefly, cells were fixed for 15 min in constant rotation in 1 mL 4% paraformaldehyde solution (Ted Pella, Reeding CA, cat. no. 18501; in 0.1 M sucrose), followed by 2 washes in 1 mL KPO_4_ and one wash with 0.1 M KPO_4_/1.2 M sorbitol at 2000*g* for 3 min at room temperature. Spheroplasts were generated by resuspending cells in 1 mL of 0.1 M KPO_4_/1.2 M sorbitol, 0.3 M β-mercaptoethanol, and 0.1 mg/mL Zymolase-100T and incubating for 12–13 min. Spheroplasts were harvested by centrifugation at 1000 rpm for 1 min at room temperature, and then washed twice with 0.1 M KPO_4_/1.2 M sorbitol. Spheroplasts were resuspended in 50 μL 0.1 M KPO_4_. Fifteen μL of cells were adhered to Teflon coated slides that were coated with 0.1% poly lysine (Epredia, Portsmouth, NH, cat. no. 86–010) and the supernatant aspirated off. The slide was immediately submerged into ice-cold methanol for 5 min, followed by submersion into room temperature acetone for 30 s, and allowed to air-dry. The cells were then blocked for 30 min with 25 μL PBS-BSA (150 μM Bovine Serum Albumin, 0.05 M KPO_4_, 0.15 M NaCl, 30 mM NaN_3_). The cells were then incubated overnight with primary antibody (1:100 rabbit anti-FLAG in PBS-BSA) in a humid chamber. Primary antibodies were aspirated off, and the slide was washed 5 times in PBS-BSA, followed by 1.25 h incubation with secondary antibody (antimouse AlexaFluor-586 1:1000) in a dark humid chamber. Secondary antibodies were then aspirated off and the slide was washed 5 times with PBS-BSA, followed by 2 washes with sterile filtered PBS. All wash volumes were 25 μL per well and all steps after addition of secondary antibodies were done in the dark. Cells were mounted with 5uL Fluoromount-G Mounting Medium with DAPI (Invitrogen, Waltham, MA, cat. no. 00–4959–52). The slides were imaged on a Zeiss LSM 800 confocal microscope at 63× magnification using the DAPI and AF586 lasers. Laser intensity was kept constant between control and (PR)_50_ samples. ZEN blue software was used for image acquisition, and the resulting images were processed using ImageJ.^57^

### Flow Cytometry and Cell Cycle Analysis

W303a yeast expressing (PR)_50_ and ccdB control were prepared for cell cycle analysis as previously described.^84^ Cell pellets were resuspended with 1.5 mL water and fixed overnight at 4 °C with 3.5 mL of 95% ethanol, added slowly. Cells were collected by centrifugation at 3000 rpm for 3 min. Pellet was washed with 25 mL of water and centrifuged again to pellet. Cell pellet was resuspended with 1.0 mL of water and transferred to a microcentrifuge tube. Cells were centrifuged at 10,000 rpm for 1 min and supernatant was discarded. Pellet was resuspended in 0.5 mL 50 mM Tris-HCl (pH 8.0) and 5 μL RNase A/T1Mix (Thermo Fisher-Scientific). Resuspended samples were incubated at 37 °C overnight. Cells were pelleted by centrifugation at 10,000 rpm for 1 min and supernatant was discarded. Pellet was resuspended in 20 μL of 10 mg/mL Proteinase K (Millipore Sigma)^85,86^ and incubated at 37 °C for 30 min. Cells were collected by brief centrifugation at 10,000 rpm, supernatant was discarded, and the pellet was resuspended in 0.5 mL of 50 mM Tris-HCl (pH 8.0).

For each sample, 50 μL of fixed cell suspension was diluted to 1.0 mL total volume using 50 mM Tris-HCl (pH 8.0) in a 1.5 mL microcentrifuge tube. For staining, 1.0 μL of 3.34 mM SYTO 9 stock solution (Invitrogen) was added, mixed thoroughly, and then incubated at 37 °C overnight in total darkness. Each sample was then lightly vortexed followed by water bath sonication for 15 s (1510 Branson Ultrasonic Bath; 40 kHz) before being analyzed using a BD Accuri C6 Sampler Plus flow cytometer. 20,000 events were collected per sample at a particle threshold of 50,000 under slow flow. FSC-H/FSC-A gating was used to select single-cell yeast populations and applied to subsequent cell cycle histograms (SYTO 9 detected in FITC channel [533/30 band-pass filter]). Data was then processed using FlowJo software.^87^

### Dot Blotting

Frozen yeast pellets were prepared as described for Western blotting preparation. Mammalian cells were lysed on ice with RIPA buffer (100 μL per 10^6^ cells) for 30 min.^88,89^ After lysis, total protein was quantified using Qubit Fluorometer 4.0 (Thermo Fisher Scientific), using a Qubit Protein Broad Range (BR) Assay Kit (Thermo Fisher Scientific, cat. no. A50668). Samples were loaded onto a nitrocellulose membrane (LI-COR Biosciences, Lincoln, NE) with a final concentration of 1 μg in 10 μL using the Bio-Dot Microfiltration Apparatus (BioRad). The membrane was stained with Revert 700 stain (LI-COR BioSciences) according to the manufacturer’s instructions and imaged using a LiCor Odyssey F imager (LI-COR BioSciences) after staining and destaining.^88,89^ Membranes were blocked using LI-COR blocking buffer (LI-COR Biosciences) for 1 h at room temperature with gentle rocking. Again, primary antibody incubations were performed at 4 °C overnight with rocking. Blots were processed using goat antimouse and goat antirabbit secondary antibodies (LI-COR Biosciences) and imaged with LiCor Odyssey F imager (LI-COR Biosciences). Protein levels were compared to the total protein stain for normalization.

### Fibroblast Cell Culture

C9ALS/FTD patient-derived fibroblasts were a gift from Rothstein (John Hopkins University). A full list of all cell lines used can be found in Table S1. Fibroblasts were cultured using DMEM with Glutamax (Gibco, Amarillo, TX, cat. no. 10–569–010) supplemented with 14% fetal bovine essence (Cytiva, Marlborough, MA, cat. no. SH3010903) and 100 U/L Penicillin-Streptomycin (Gibco Amarillo, TX, cat. no. 15–140–122) in T-75 cell culture flasks. All cells were cultured at 37 °C with 5.0% CO_2_. During passaging, 5–15 million cells were harvested by centrifugation at 400 g and 4 °C for 5 min, followed by washing with 10 mL sterile DPBS (Gibco, Amarillo, TX, cat. no. 14190144) two times. The resulting pellet was flash frozen in liquid nitrogen N_2_ and stored at −80 °C. Each line was harvested at least three separate times. The morphology of the fibroblasts was checked at each feeding to ensure purity.

### Induced Pluripotent Stem Cell Culture

C9ALS/FTD patient-derived induced pluripotent stem cells (iPSCs) were obtained from the NINDS Human Cell and Data Repository at Cedars-Sinai. A full list of all cell lines used can be found in Table S1. iPSCs were cultured on plates treated with Matrigel Matrix (Corning, Corning, NY, cat. No. 35427) using mTeSR1 (StemCell, Vancouver, Canada, cat. No. 85851) culture medium supplemented with Y-27632 (StemCall, Vancouver Canada, cat. No. 72302) and 100 U/L Penicillin-Streptomycin (Gibco, Amarillo, TX, cat. No. 15–140–122). All cells were cultured at 37 °C with 5.0% CO_2_ gas. Cells were passaged with 1 U/mL Dispase (StemCell, Vancouver, Canada, cat. No. 07923) at ∼75% confluency. During passaging, 10–20 million cells were harvested by centrifugation at 400*g* and 4 °C for 5 min, followed by washing with 10 mL sterile DPBS (Gibco, Amarillo, TX, cat. no. 14190144) two times. The resulting pellet was flash frozen in liquid N_2_ and stored at −80 °C. Each line was harvested at least three separate times. The morphology of the iPSCs were checked at each feeding to ensure purity.

### Histone Isolation

Histones were isolated from c9ALS/FTD fibroblasts and iPSCs by acid extraction.^90,91^ Briefly, cell pellets were lysed in Nuclear Extraction Buffer (NIB, 15 mM Tris-HCl pH 7.5, 15 mM NaCl, 60 mM KCl, 5 mM MgCl_2_, 1 mM CaCl_2_, 250 mM sucrose, 1 mM DTT, 0.5 mM AEBSF, 5 nM Microcystin) and 0.2% NP-40 for 15 (fibroblasts) or 10 (iPSCs) minutes on ice at a 10:1 vol/packed cell pellet vol ratio, followed by three washes with NIB at 1000*g* for 5 min at 4 °C at a 10:1 vol/vol ratio. The pellet was then resuspended in a 5:1 vol/vol ratio of 0.4 N H_2_SO_4_ and incubated with constant rotation for 4 h at 4 °C. Cellular debris was removed by centrifugation at 3400*g* for 5 min at 4 °C and transferring the histone-containing supernatant to a fresh tube, twice. Histones were then precipitated with 100% TCA overnight at 4 °C. Histones were pelleted by centrifugation at 3400*g* for 5 min at 4 °C, then followed by a wash with 0.1% HCl in acetone, followed by a final wash in pure acetone. The pellet was allowed to air-dry completely and was then dissolved in nuclease-free water. Protein concentration was measured with a Bradford Assay (Bio-Rad, Hercules, CA, cat. no. 5000205). Samples were standardized to 0.07 μg/μL. Histone purity was assessed by Coomassie (Bio-Rad, Hercules, CA, cat. no. 1610400) staining following SDS-PAGE on an 18% gel. Histone post-translational modification levels were then measured by Western blotting as described above.

### Data and Statistical Analysis

Densitometric analysis of Western blots was performed using Image Studio Software (LI-COR Biosciences). The Draw Rectangle tool was used to select the area of the protein bands on each individual channel imaged (700 and 800) to provide raw signal values. An additional area of the background was quantified, and this signal was removed from the signals of all samples to account for background noise. Signals obtained for histone modifications were normalized to their respective histone H3 signals (modification signal/H3 Total signal = Relative Density). Anti-H3K36me2 (Abcam) produces two bands, the topmost band at 17 kDa was used for quantifications, per the manufacturer’s recommendation. Relative density values were then used for statistical analysis. RNA concentrations were reported as raw values given by the Qubit in μg/mL. Quantification of aggregates per cell from immunofluorescence experiments was conducted using the AggreCount Macro in ImageJ as described by Klickstein et al. Settings were kept consistent among all images.^92^ Statistical analysis of data was performed in GraphPad Prism (GraphPad Software, Boston, MA). Significant differences between sample groups were determined using two-tailed Welch’s *t*-test with *p* = 0.05 as the cutoff for significance. All data were analyzed with the Robust Regression Outlier Test (ROUT) to identify outliers with a *Q* = 1%.^93^ A large number of individual *t* tests significantly increases the chance of a false finding of significance, thus a two-stage step-up method of Benjamini, Krieger, and Yekutieli False Discovery Rate approach was used. Error bars represent the standard deviation (SD) calculated from the fold changes obtained.

## Conclusions

We find that (PR)_50_ proteinopathy is linked to changes in the histone PTM landscape. Using yeast as a discovery platform, we find that overexpression of (PR)_50_ is connected to increases in the levels of various histone PTMs, including a novel increase in H3S10ph levels. Knockdown of Ipl1 in yeast led to both restoration of normal H3S10ph levels as well as an overall rescue from (PR)_50_’s toxic effects in yeast. This ultimately suggests that presence of poly-PR disrupts normal histone modifying enzyme function. Recapitulating our yeast results, we have also identified a significant increase in the levels of H3S10ph in c9ALS/FTD patient derived fibroblasts and iPSCs, further suggesting a role for H3S10ph dysregulation in the etiology of c9ALS/FTD. Future work aimed at elucidating the mechanisms connecting (PR)_50_ aggregation to the epigenome will likely yield novel avenues for pharmaceutical intervention that will allow us to bypass the detrimental effects of protein aggregation in neurodegeneration.

## Supplementary Material



## Data Availability

All data is provided within the text and Supporting Information.

## Notes

Further (PR)_50_ expression verification in W303 yeast, (PA)_50_, (GA)_50_, (GR)_100_ blots probing for DPR expression and H3S10ph level changes, linear range assays for select antibodies, and other Western blots for histone PTMs displaying unchanged levels in (PR)_50_ yeast, (PR)_50_ flow cytometry data, TDP-43 overexpression data, C9 ALS/FTD fibroblast and C9ALS/FTD patient-derived iPSC data, lists of fibroblast and iPSC lines used, as well as antibodies used in this study are also included (PDF)
