# Supplementary material for: C9orf72 Dipeptide Repeat Proteinopathy Is Linked to Increased Histone H3 Phosphorylation on Serine 10
Source: ACS Omega. 2025 Oct 9;10(41):48395–411. doi: 10.1021/acsomega.5c05836 (PMC12547607; doi:10.1021/acsomega.5c05836)
Supplement: Supplementary file 1 [file ao5c05836_si_001.pdf]

C9orf72 dipeptide repeat proteinopathy is linked to increased histone H3 phosphorylation on serine 10

Samantha N. Cobos[1,2,3#], Raven M.A. Fisher[1,4#], Seth A. Bennett[1,4,5], Chaim Janani[1], David K. Dansu[4,6,7], Matthew M. Cleere[8,9], Arefa Yeasmin [1,8], Gabriel Cruz[1], Sidra Qureshi[1], William Villasi[1], Rania Frederic[1], Kyle Chen[1], Melagras Mirzakandova[1], George Angelakakis[1], Elizaveta Son[1], Andrew Elgendy[1], and Mariana Torrente[1,2,4,8]*

[1]Department of Chemistry and Biochemistry, Brooklyn College, Brooklyn, New York, 11210, USA
[2]Ph.D. Program in Chemistry, The Graduate Center of the City University of New York, New York, New York, 10016, USA
[3] Present Address: Department of Neurobiology and Behavior, Stony Brook University, Stony Brook, NY, 11794, USA
[4]Ph.D. Program in Biochemistry, The Graduate Center of the City University of New York, New York, New York, 10016, USA
[5] Present Address: Regeneron Pharmaceuticals, Inc., Rensselaer, NY 12144, USA.
[6]Neuroscience Initiative, Advanced Science Research Center, CUNY, New York, NY 10031, USA
[7] Present Address: Regeneron Pharmaceuticals, Inc., Tarrytown, NY 10591, USA.
[8]Ph.D. Program in Biology, The Graduate Center of the City University of New York, New York, New York, 10016, USA
[9]Structural Biology Initiative, Advanced Science Research Center, CUNY, New York, NY 10031, USA

*Corresponding Author: mariana.torrente@brooklyn.cuny.edu
#Authors contributed equally to this work

Supporting Information

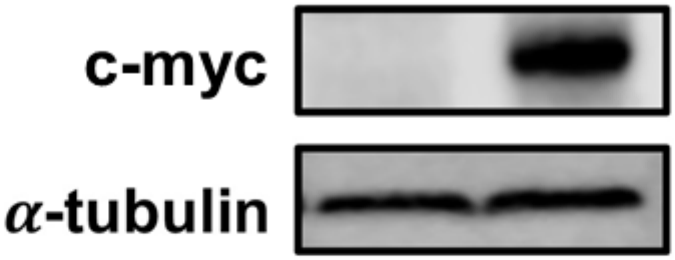

Figure S1. $(PR)_{50}$ overexpression is verified in W303 yeast with western blotting. Representative blot confirming $(PR)_{50}$ in $(PR)_{50}$ yeast compared to the vector control (ccdB). A c-myc antibody was used to detect the c-myc $(PR)_{50}$, with α-tubulin as the loading control. (n=3).

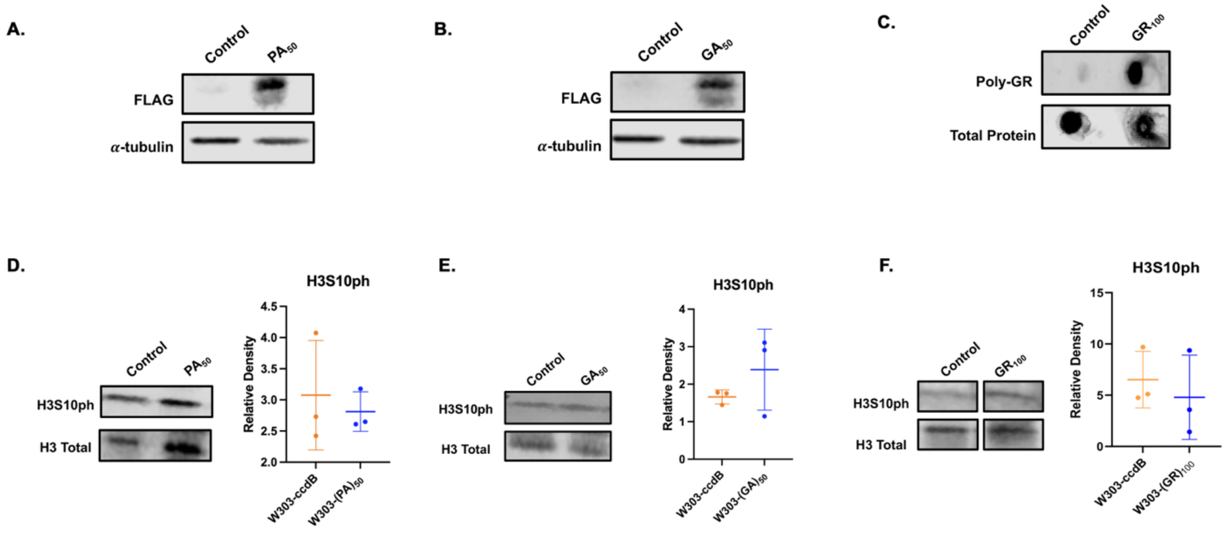

Figure S2. $(PA)_{50}$, $(GA)_{50}$, $(GR)_{100}$ overexpression does not impact H3S10ph levels. Representative blots confirming $(PA)_{50}$, $(GA)_{50}$, $(GR)_{100}$ expression in (A) $(PA)_{50}$ yeast, (B) $(GA)_{50}$ yeast, (C) $(GR)_{100}$ yeast. Representative blots showing the levels of H3S10ph in (D) $(PA)_{50}$ yeast, (E) $(GA)_{50}$ yeast, (F) $(GR)_{100}$ yeast compared to controls. Column scatterplots quantify the relative densities of raw histone PTM signal to histone H3 total signal in DPR overexpression yeast compared to controls. Each point in the graph represents a separate experiment with a different biological replicate. (n = 3).

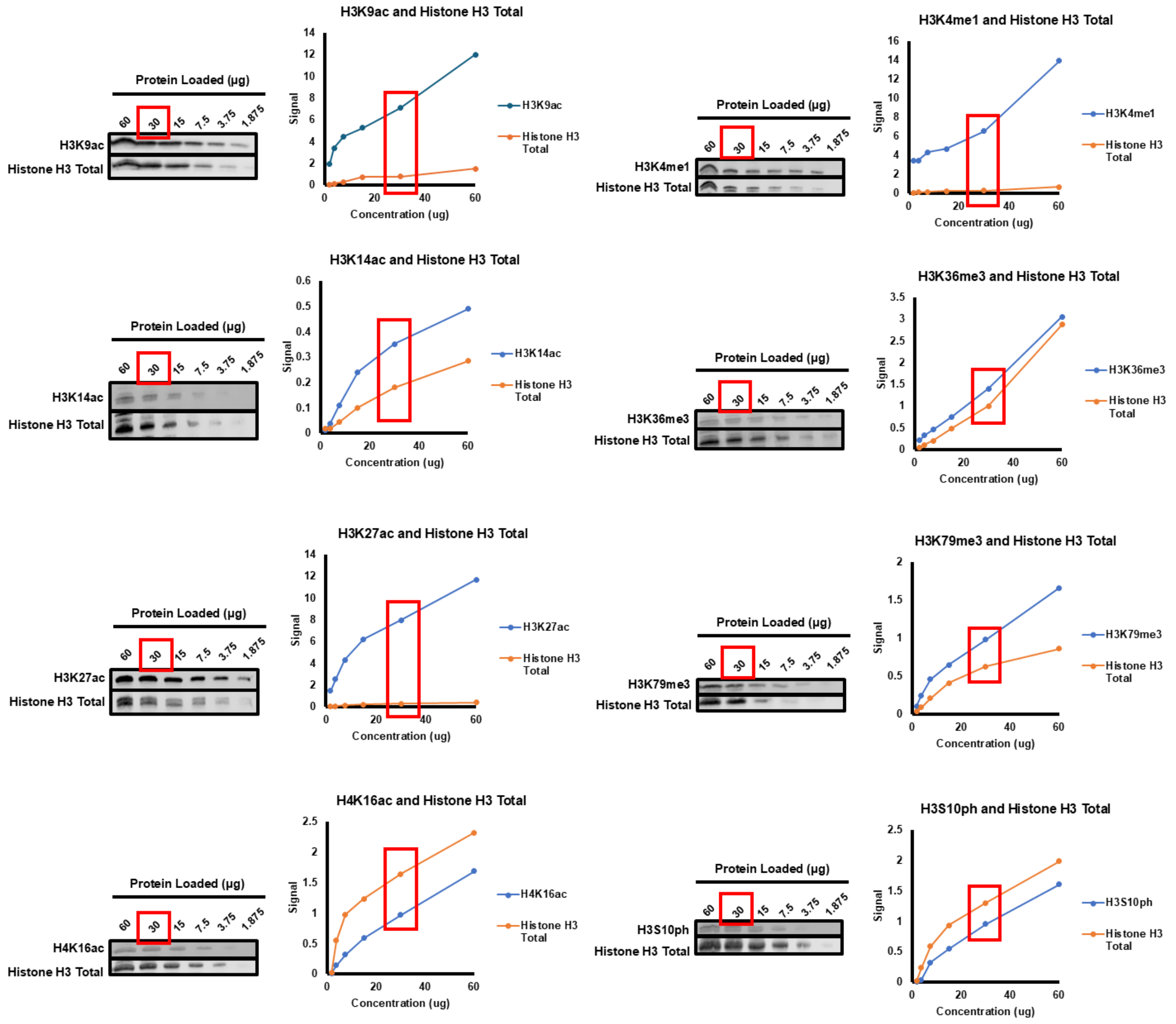

Figure S3. Linear range of detection for select antibodies. Varying protein amounts of control yeast lysate were assayed with α-H3K9ac, α-H3K14ac, α-H3K27ac, α-H4K16ac, α-H3K4me1, α-H3K36me3, α-H3K79me3, and α-H3S10ph antibodies. α-Histone H3 Total was used as a loading control. Graphs show the amount of protein loaded (μg) vs. signal intensity. Red boxes highlight the amount of protein loaded in all experiments.

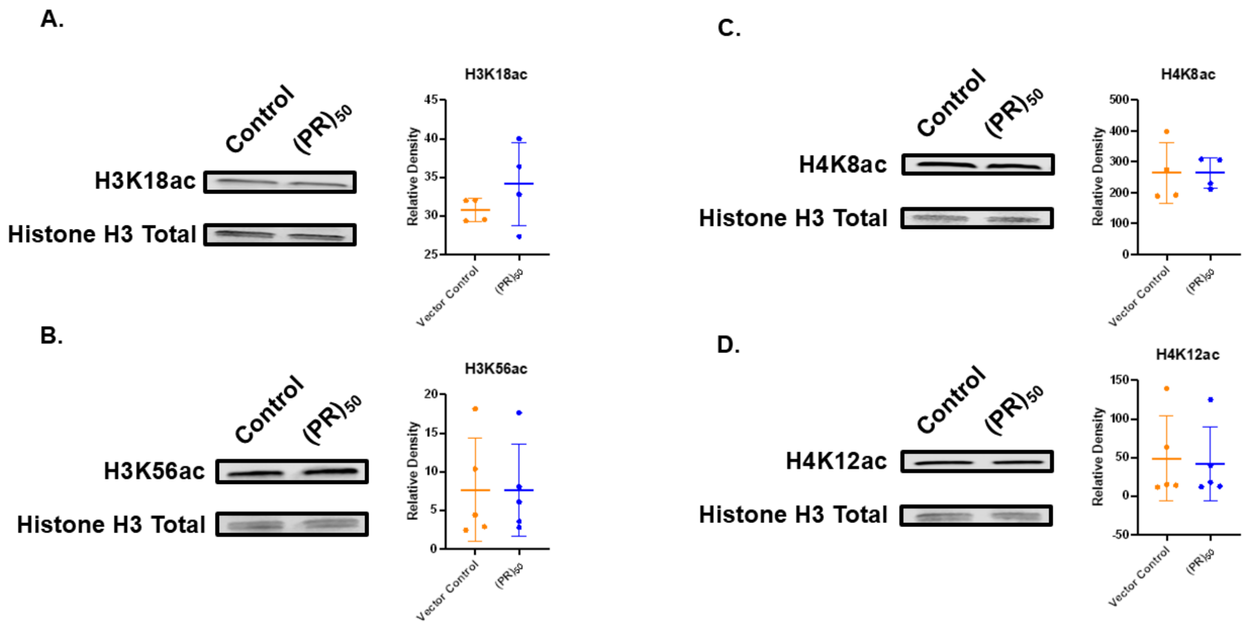

Figure S4. $(PR)_{50}$ overexpression does not impact acetylation of H3K18, H3K56, H4K8, and H4K12. Representative blots showing the levels of (A) H3K18ac, (B) H3K56ac, (C) H4K8ac, and (D) H4K12ac in $(PR)_{50}$ yeast compared to controls. Column scatterplots quantify the relative densities of raw histone PTM signal to histone H3 total signal in $(PR)_{50}$ yeast compared to controls. Each point in the graph represents a separate experiment with a different biological replicate. (n = 4-5).

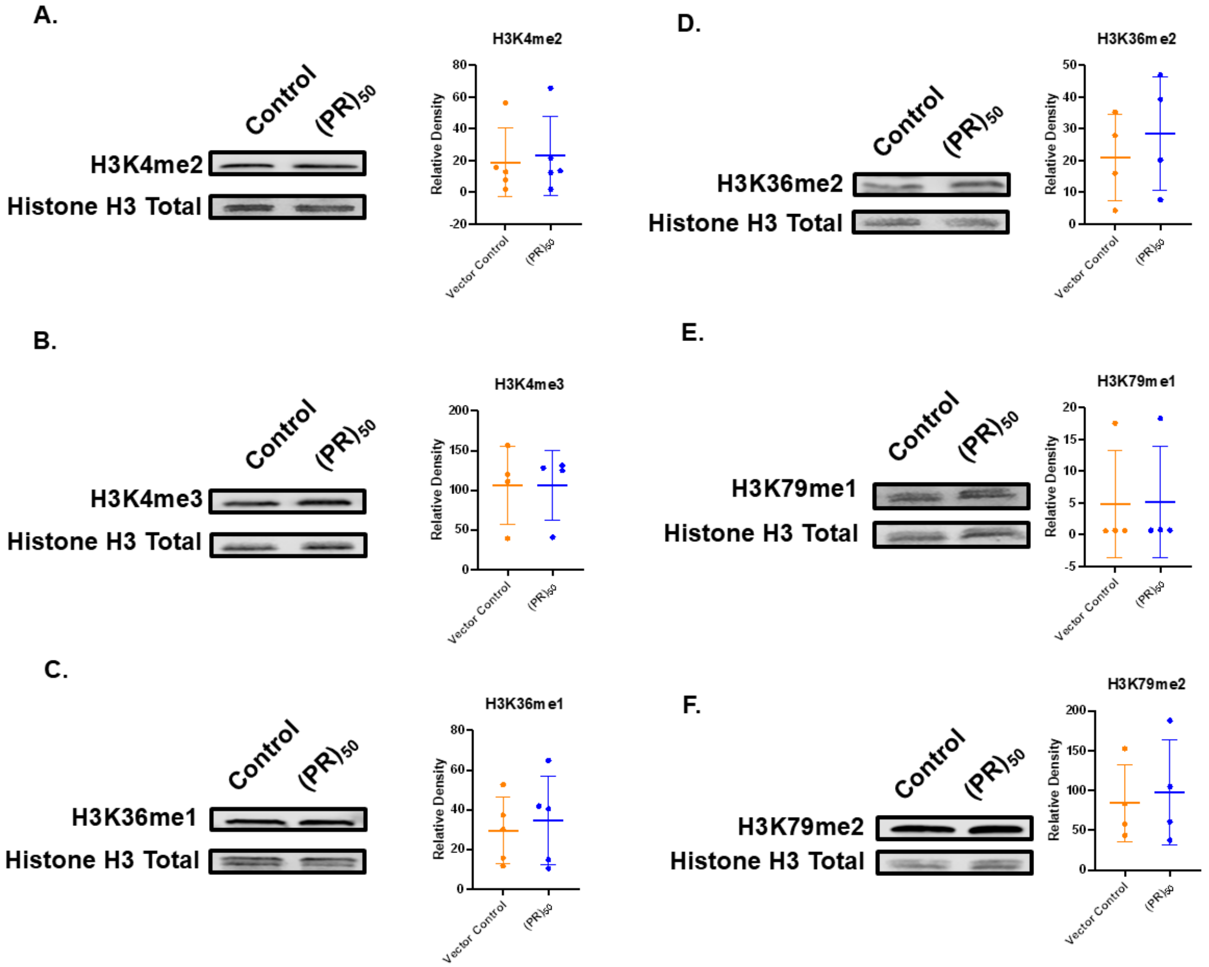

Figure S5. $(PR)_{50}$ overexpression is not linked to changes in several histone H3 and H4 methylation marks. Representative blots showing the levels of (A) H3K4me2, (B) H3K4me3, (C) H3K36me1, (D) H3K36me2, (E) H3K79me1, and (F) H3K79me2 in $(PR)_{50}$ yeast compared to controls. Column scatterplots quantify the relative densities of raw histone PTM signals to histone H3 total signals. $(PR)_{50}$ yeast compared to controls. Each point in the graph represents a separate experiment with a different biological replicate. (n = 4-5).

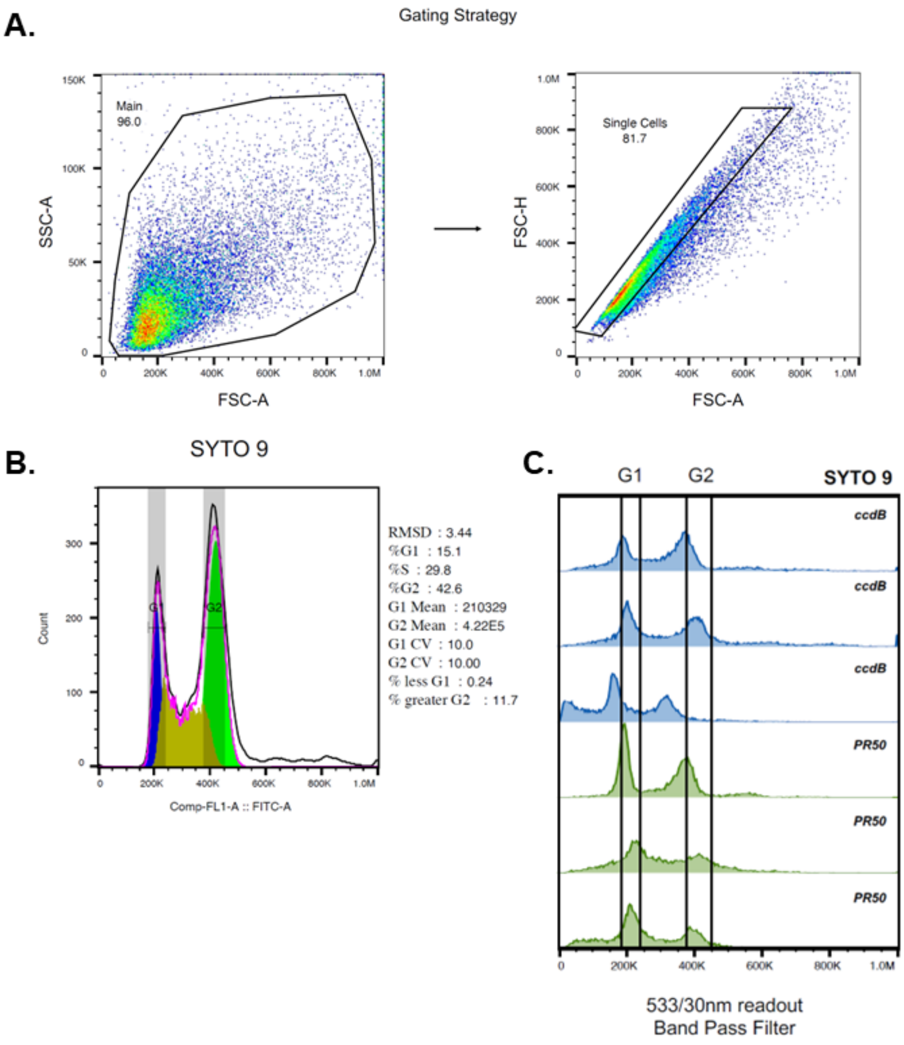

Figure S6. (PR)$_{50}$ overexpression does not lead to cell cycle arrest. (A) Density plots of gating strategy as both SSC-A/FSC-A and FSC-H/FSC-A data. (B) Plot demonstrating FlowJo Univariate cell cycle modeling using SYTO 9 dye. (C) Raw flow cytometry data obtained from each individual experiment. Each curve represents one biological replicate. (n=3).

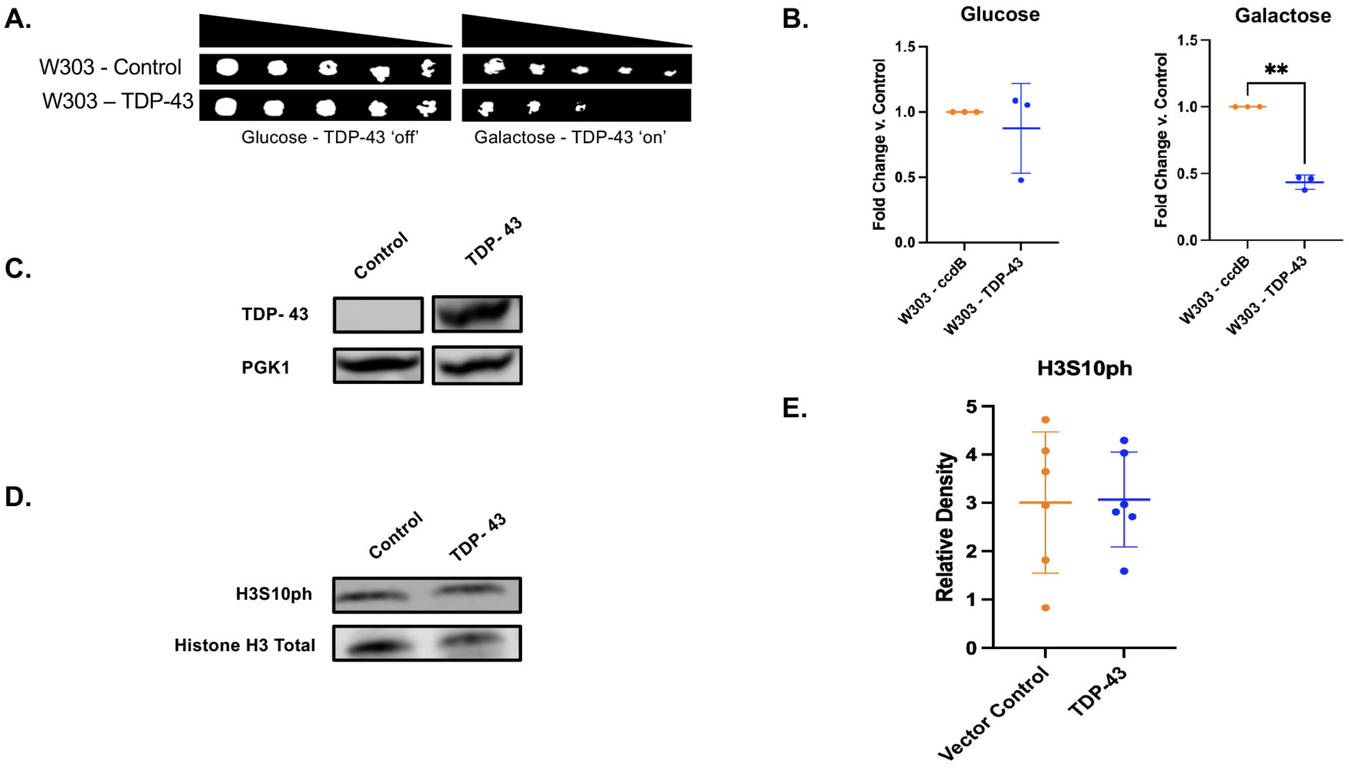

Figure S7. TDP-43 overexpression is not linked to H3S10ph changes. (A) Solid media growth assay depicting the toxicity of TDP-43 overexpression in W303 yeast. (B) Column scatterplots show densitometric analysis of cell density of TDP-43 yeast compared to control on both glucose and galactose plates in (A)
** = $p < 0.001$. (n=3). (C) Representative blots verifying expression of TDP-43 in W303 yeast compared to control. (D) Representative blot showing the levels of H3S10ph in W303 TDP-43 yeast compared to control. (E) Column scatterplots quantify the relative densities of raw H3S10ph signal to histone H3 total signal in TDP-43 yeast compared to control. Each point in the graph represents a separate experiment with a different biological replicate. (n = 6).

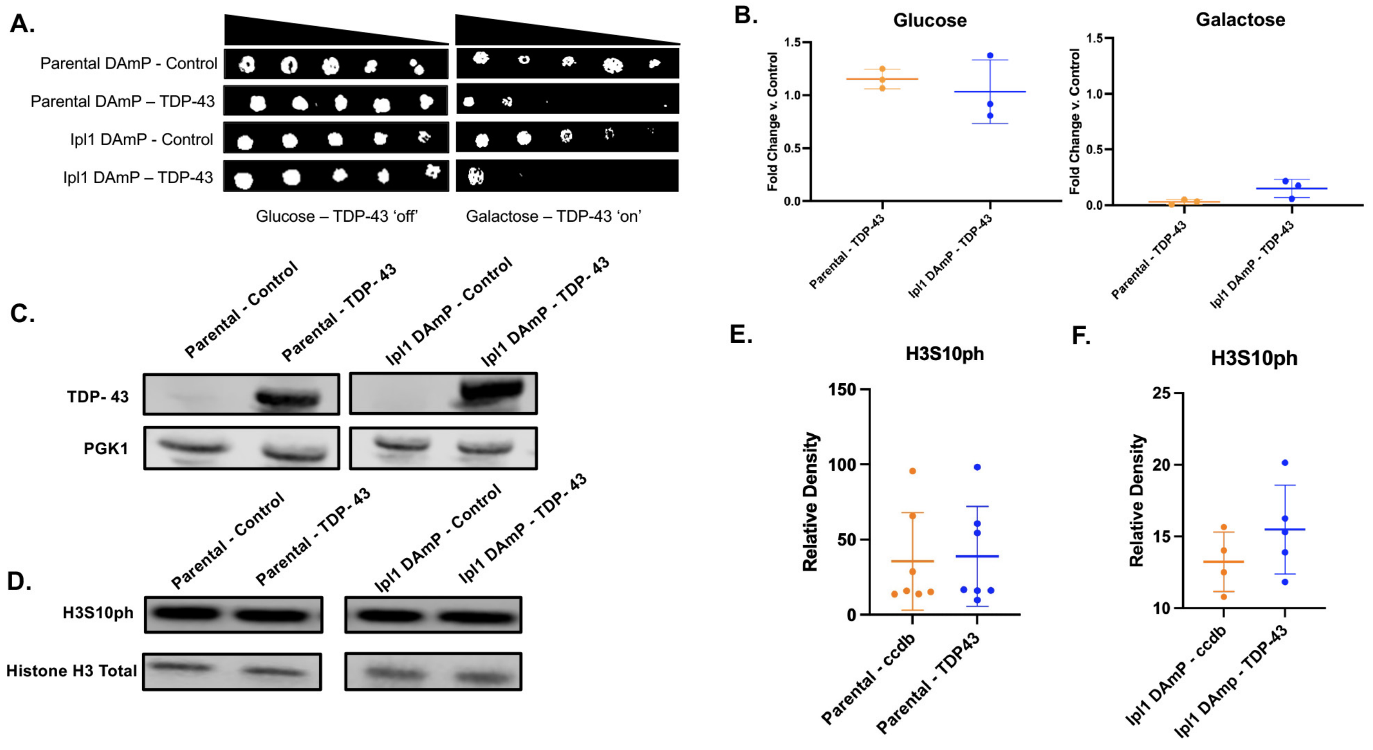

Figure S8. mRNA perturbation of Ipl1 does not affect H3S10ph levels in the context of TDP-43 overexpression. (A) Solid media growth assay verifying the toxicity of TDP-43 overexpression in parental BY4741 and Ipl1 DAmP yeast. (B) Column scatterplots show densitometric analysis of cell density of TDP-43 in parental BY4741 and Ipl1 DAmP yeast compared to control on glucose and galactose plates in (A) (n=3). (C) Representative blots verifying expression of TDP-43 in parental BY4741 and Ipl1 DAmP yeast compared to control. (D) Representative blots showing the levels of H3S10ph in parental BY4741 and Ipl1 DAmP yeast expressing control and TDP-43 plasmids. (E) Column scatterplots quantify the relative densities of raw H3S10ph signal to histone H3 total signal in parental control and TDP-43 yeast. (n = 7) (F) Column scatterplots quantify the relative densities of raw H3S10ph signal to histone H3 total signal in Ipl1 DAmP control and TDP-43 yeast. Each point in the graph represents a separate experiment with a different biological replicate. (n = 4-6).

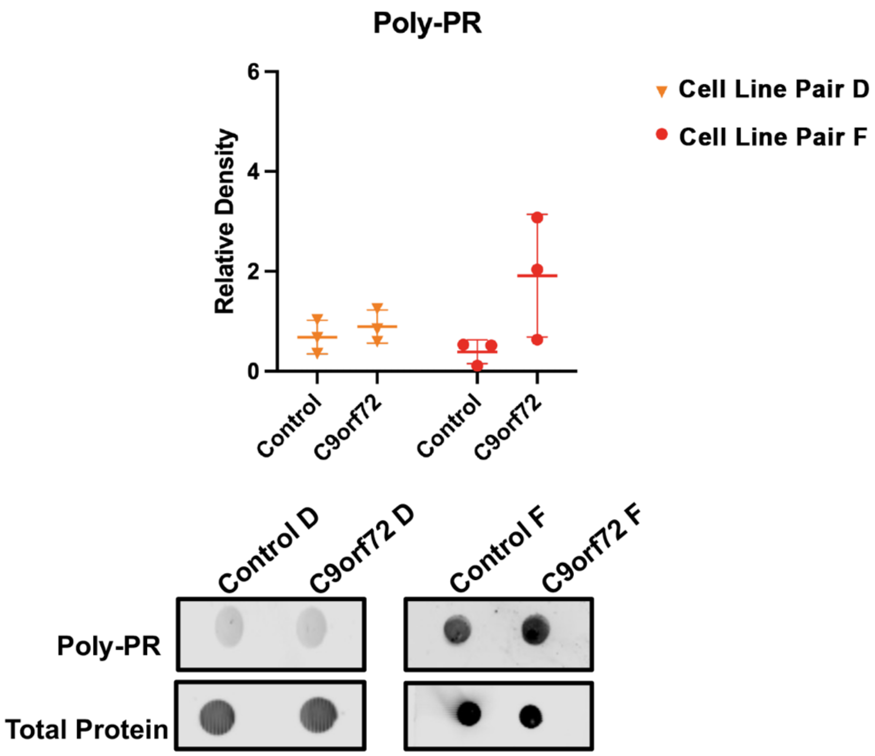

Figure S9. DPR levels in c9 ALS fibroblasts. Representative dot blots probing for Poly-PR levels in whole cell lysates from c9 ALS patient-derived fibroblasts. Column scatterplots quantify the relative densities of raw Poly-PR signal to Total Protein. Each point in the graph represents a separate experiment with a different biological replicate. (n = 3)

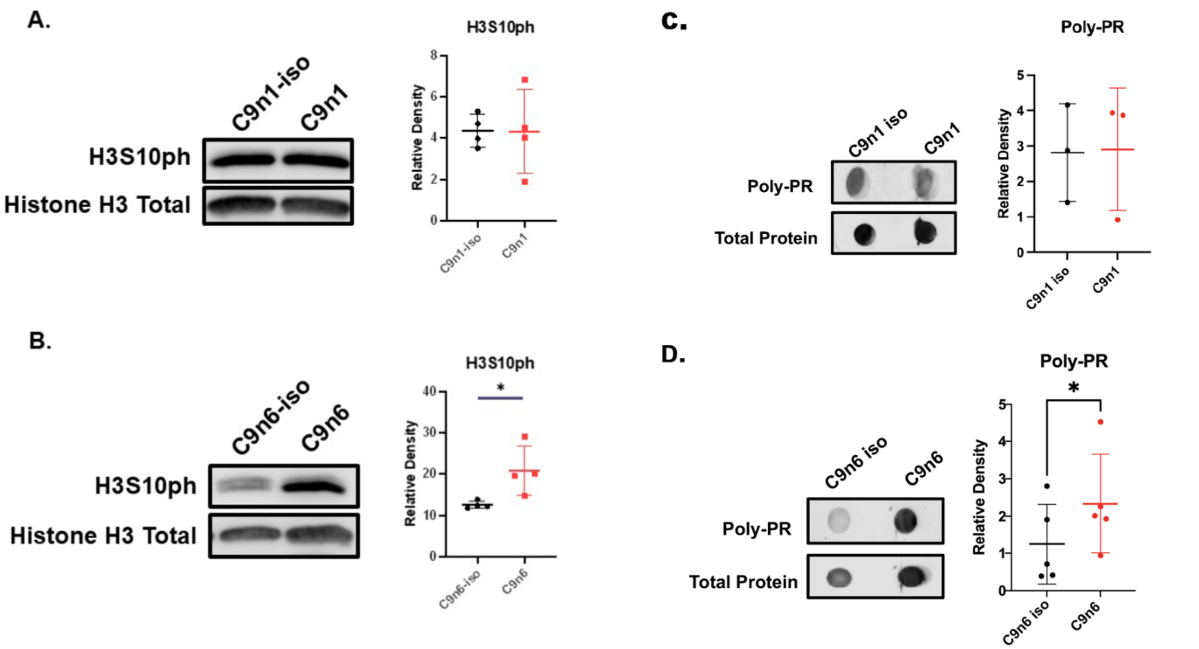

Figure S10. C9ALS/FTD patient-derived induced pluripotent stem cells display an increase in levels of H3S10ph and Poly-PR. Representative blots depicting changes in levels of H3S10ph in (A) C9n1 and (B) C9n6 iPSC lines derived from male c9ALS/FTD patients, along with isogenic, CRISPR-corrected controls. Representative dot blots depicting Poly-PR levels in (C) C9n1 and (D) C9n6 iPSC lines compared to isogenic, CRISPR-corrected controls. Column scatterplots quantify the relative densities of Poly-PR signal to total protein stain. Each point in the graph represents a separate experiment with a different biological replicate. (n = 3-5), * = $p \leq 0.05$.

Table S1. List of fibroblast and iPSC lines used in this study.

Line	Source	Institutional ID	Race	Gender	Passage #	Age of onset	Age of Biopsy	Cell Type
**A**	Johns Hopkins University	33	White	Male	5	62	65	Fibroblast
	Johns Hopkins University	82	White	Male	4	N/A	61	Fibroblast
**B**	Johns Hopkins University	78	White	Male	5	57	58	Fibroblast
	Johns Hopkins University	82	White	Male	4	N/A	61	Fibroblast
**C**	Johns Hopkins University	34	White	Female	5	63	65	Fibroblast
	Johns Hopkins University	31	White	Female	7	N/A	70	Fibroblast
**D**	Johns Hopkins University	57	White	Female	2	56	59	Fibroblast
	Johns Hopkins University	31	White	Female	7	N/A	70	Fibroblast
**E**	Johns Hopkins University	15	White	Female	8	53	50	Fibroblast
	Johns Hopkins University	3	White	Female	5	N/A	39	Fibroblast
**F**	Johns Hopkins University	92	White	Male	8	48	51	Fibroblast
	Johns Hopkins University	88	White	Male	2	N/A	39	Fibroblast
**C9n1**	Cedars-Sinai RMI iPSC Core	CS29iALS-n1N	White	Male	25	46	46	iPSC
	Cedars-Sinai RMI iPSC Core	CS29iALS-n1N-iso	White	Male	25	46	46	iPSC
**C9n6**	Cedars-Sinai RMI iPSC Core	CS52iALS-n6A	White	Male	26	45	48	iPSC
	Cedars-Sinai RMI iPSC Core	CS52iALS-n6A-iso	White	Male	26	45	48	iPSC

Table S2. List of antibodies used in this study.

Antibody	Manufacturer	Catalog #	Dilution
H3 Total	Abcam	ab24834	1:2,000
H3K4me1	Abcam	ab8895	1:500
H3K4me2	Abcam	ab7766	1:1,000
H3K4me3	Abcam	ab8580	1:500
H3K9ac	Abcam	ab10812	1:500
H3K14ac	Millipore	07-353	1:5,000
H3K18ac	Abcam	ab1191	1:1,000
H3K27ac	Abcam	ab45173	1:500
H3K36me1	Abcam	ab9048	1:1,000
H3K36me2	Abcam	ab9049	1:1,000
H3K36me3	Abcam	ab9050	1:1,000
H3K56ac	ActiveMotif	39281	1:5,000
H3K79me1	Millipore	ABE213	1:500
H3K79me2	Abcam	ab3594	1:1,000
H3K79me3	Abcam	ab2621	1:1,000
H3S10ph	Abcam	ab5176	1:1,000
H4K8ac	Abcam	ab15823	1:1,000
H4K12ac	Abcam	ab46983	1:5,000
H4K16ac	Abcam	ab109463	1:2,000
TDP-43	Proteintech	10782-2-AP	1:1,000
C9orf72/C9RANT (Poly-PR)	Millipore	ABN1354	1:1,000
C9orf72/C9RANT (Poly-GR)	Proteintech	23978-1-AP	1:1000
GAPDH	Cell Signaling	97166S	1:1000
Phosphoglycerate Kinase 1	Thermo	459250	1:2,000
Alpha Tubulin	Abcam	184966	1:10,000
c-Myc	Thermo	MA1-980	1:1,000
Goat Anti Rabbit Secondary	Licor	A11008	1:20,000
Goat Anti Mouse Secondary	Licor	A11004	1:10,000
